# How Regiochemistry Influences Aggregation Behavior and Charge Transport in Conjugated Organosulfur Polymer Cathodes for Lithium–Sulfur Batteries

**DOI:** 10.1021/acsnano.3c01523

**Published:** 2023-04-04

**Authors:** Yannik Schütze, Diptesh Gayen, Karol Palczynski, Ranielle de Oliveira Silva, Yan Lu, Michael Tovar, Pouya Partovi-Azar, Annika Bande, Joachim Dzubiella

**Affiliations:** 1 Research Group for Simulations of Energy Materials, Helmholtz-Zentrum Berlin für Materialien und Energie GmbH, Hahn-Meitner-Platz 1, 14109 Berlin, Germany; 2 Theoretical Chemistry, Institute of Chemistry and Biochemistry, Freie Universität Berlin, Arnimallee 22, 14195 Berlin, Germany; 3 Applied Theoretical Physics - Computational Physics, Physikalisches Institut, Albert-Ludwigs-Universität Freiburg, Hermann-Herder-Straße 3, 79104 Freiburg, Germany; 4 Department Electrochemical Energy Storage, Helmholtz-Zentrum Berlin für Materialien und Energie GmbH, Hahn-Meitner-Platz 1, 14109 Berlin, Germany; 5 Institute of Chemistry, University of Potsdam, Am Neuen Palais 10, 14469 Potsdam, Germany; 6 Department Structure and Dynamics of Energy Materials, Helmholtz-Zentrum Berlin für Materialien und Energie GmbH, Hahn-Meitner-Platz 1, 14109 Berlin, Germany; 7 Institute for Chemistry, Martin Luther Universität Halle-Wittenberg, Von-Danckelmann-Platz 4, 06120 Halle (Saale), Germany; 8 Theory of Electron Dynamics and Spectroscopy, Helmholtz-Zentrum Berlin für Materialien und Energie GmbH, Hahn-Meitner-Platz 1, 14109 Berlin, Germany

**Keywords:** lithium−sulfur battery, conjugated polymer, regularity, self-assembly, charge transport, molecular dynamics simulations, X-ray diffraction

## Abstract

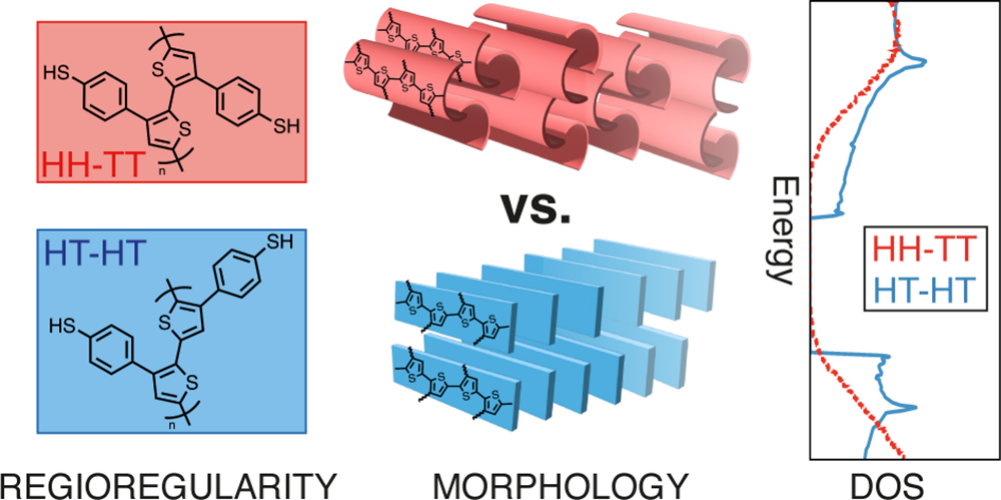

For lithium–sulfur (Li–S) batteries to become competitive, they require high stability and energy density. Organosulfur polymer-based cathodes have recently shown promising performance due to their ability to overcome common limitations of Li–S batteries, such as the insulating nature of sulfur. In this study, we use a multiscale modeling approach to explore the influence of the regiochemistry of a conjugated poly(4-(thiophene-3-yl)benzenethiol) (PTBT) polymer on its aggregation behavior and charge transport. Classical molecular dynamics simulations of the self-assembly of polymer chains with different regioregularity show that a head-to-tail/head-to-tail regularity can form a well-ordered crystalline phase of planar chains allowing for fast charge transport. Our X-ray diffraction measurements, in conjunction with our predicted crystal structure, confirm the presence of crystalline phases in the electropolymerized PTBT polymer. We quantitatively describe the charge transport in the crystalline phase in a band-like regime. Our results give detailed insights into the interplay between microstructural and electrical properties of conjugated polymer cathode materials, highlighting the effect of polymer chain regioregularity on its charge transport properties.

Lithium–sulfur (Li–S) batteries are among the most promising next-generation energy storage systems that have the potential to surpass traditional lithium-ion batteries in terms of both energy density and cost.^1−3^ These batteries are composed of a lithium anode and a sulfur cathode and operate through a redox reaction in which lithium ions are transferred between the two electrodes. This reaction allows Li–S batteries to store more energy per unit weight than lithium-ion batteries, making them an attractive option for a wide range of applications.^4,5^ However, despite their potential advantages, Li–S batteries have faced several challenges in terms of their performance and stability, such as the insulating nature of the elemental sulfur^6^ and the shuttle effect of dissolvable lithium polysulfides.^7^

Conjugated polymers are a class of polymeric materials that exhibit alternating single and double carbon bonds in their main chain, which gives them characteristic electrical, optical, and mechanical properties.^8−11^ In the context of Li–S batteries, conjugated polymers can be used as cathode material to improve the electrical conductivity^12−14^ of the sulfur cathode and to prevent the dissolution of the active materials by forming strong chemical bonds with sulfur.^15−19^ Among these, thiol-containing polymers are one example where the −SH groups can be cross-linked with sulfur via covalent bonding.^20−22^

In a recent study of ours, we presented a fabrication strategy to construct a binder- and carbon additive-free organosulfur cathode based on a thiol-containing conducting polymer poly(4-(thiophene-3-yl)benzenethiol) (PTBT).^22^ The PTBT features the polythiophene main chain as a highly conducting framework and the benzenethiol side chain to copolymerize^23,24^ with sulfur and form a cross-linked organosulfur polymer. We have been able to show the significant fixing effect of the sulfur species by operando X-ray imaging. This synthesis approach maintains the conductivity and flexibility of the polymer framework and therefore seems promising to overcome typical drawbacks of Li–S batteries. In a further study, we used a combination of *first-principles* computational methods and statistical mechanics to explore the structural characteristics of the initial state of a vulcanized PTBT polymer.^25^ Our calculations showed that the main reaction of the vulcanization process leads to high-probability states of sulfur chains cross-linking TBT units belonging to different polymer backbones, with a dominant sulfur chain length of 5 atoms. Similar results have been reported for different sulfur/carbon copolymers.^14,26^

The morphology of a polymer can greatly impact its conductive behavior. In general, polymers with more ordered and aligned structures allow for more efficient transport of charges and tend to have higher conductivity than those with disordered structures. The morphology of the polymer can be controlled through various synthesis techniques.^27−29^ Understanding the relationship between morphology and conductivity can allow for the design of polymers with improved conductive behavior.^30−32^ Thiophene-based conjugated polymers such as the well-known poly(3-hexylthiophene) (P3HT),^33−36^ poly(2,5-bis(3-alkylthiophen-2-yl)thieno[3,2-*b*]- thiophene) (PBTTT),^37−39^ or poly-3,4-ethylendioxythiophen (PEDOT)^40−42^ have been extensively studied over the last decades to demonstrate morphology-transport relationships, but many fundamental questions in this field of polymeric organic semiconductors remain unanswered. Furthermore, it is often not possible to directly use the theoretical description of similar thiophene-based polymers when investigating a different material without introducing errors. Therefore, special care must be taken to describe the PTBT’s microstructure accurately.

One important factor influencing the charge transport properties of all of these systems is the regioregularity of their backbones.^43−47^ In order to elucidate this relationship for our PTBT polymer from a theoretical point of view, the knowledge of an accurate atomistic model of the material is crucial for a structural description and the calculation of its electronic properties. In this work, we employ classical molecular dynamics (MD) simulations to explore the self-assembly process of conjugated PTBT chains of different backbone regularities. To validate our structure predictions, we compare experimental X-ray diffraction (XRD) measurements of the electropolymerized PTBT polymer with simulated diffraction patterns. Taking the generated structures as input, we then use electronic structure theory to investigate how the structural changes of the polymer influence its electronic and charge transport properties. Finally, we employ Boltzmann transport and deformation potential theory as a quantitative approach to estimating the intrinsic transport limit of the PTBT polymer. Our theoretical multiscale approach, combined with the experiments, allows us to precisely describe the interplay between polymer regularity, structural morphology, and charge transfer properties.

## Results and Discussion

### Aggregation Behavior of Regioregular PTBT Polymer Chains

**Figure 1:**
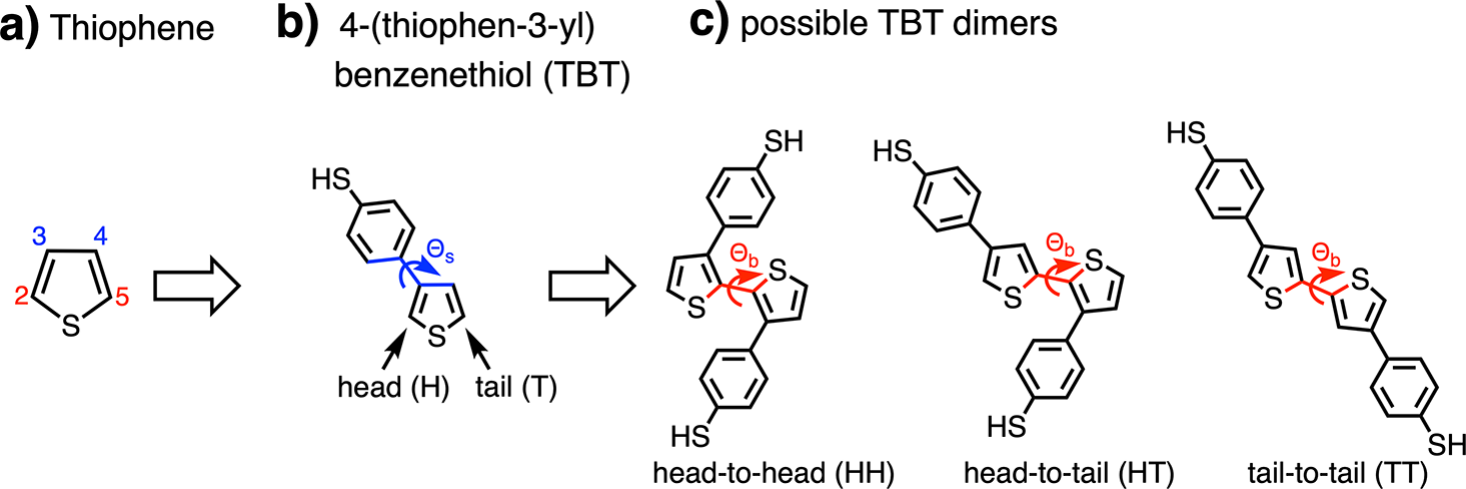
(a) Binding sites of the heterocyclic thiophene ring. The labels on the α-positions (2, 5) are colored in red, and those at the β-positions (3, 4) in blue. (b) The TBT monomer with the substituted benzenethiol at the β-position of the thiophene ring. The side chain dihedral Θ_s_ is colored in blue. (c) Illustration of the three possible connections, HH, HT, and TT, between two asymmetric TBT units. The backbone dihedral Θ_b_ is colored in red for the three structures.

In a recent study of ours,^48^ we investigated the conformational behavior of a single oligomeric PTBT chain in solution by means of MD simulations. Due to the conjugated nature of our system, one of the most important terms to be considered when evaluating the force field is the energetic profile governing the dihedral dynamics between neighboring monomers. The electropolymerization of TBT monomers results in the formation of conjugated PTBT polymer chains. The polymerization of the five-membered heterocyclic thiophene ring can take place through bonding at the α- or β- positions (2- or 3-positions, cf. Figure 1a). In the TBT monomer, the β-position is substituted with a benzenethiol group. It is well established that the α-position is the most reactive position in the polymerization of 3-substituted thiophene monomers, leading to the dominance of α,α-linkages in the resulting polymer. As a result, the long PTBT polymer chains that form during polymerization will likely have thiophene rings as the main-chain backbones with benzenethiol groups as the lateral chains.^49−52^

A key feature of the TBT monomer is the breaking of reflection symmetry between each end of the molecule (along the direction of the polymer backbone). This intrinsic asymmetry allows for three possible connections, head-to-head (HH), head-to-tail (HT), and tail-to-tail (TT), between two TBT repeat units (cf. Figure 1c). The general terms ‘head’ and ‘tail’ distinguish the substituted groups at the β-positions of consecutive thiophene rings (here, the benzenethiol group is depicted as a head (H), and the hydrogen at the 4-position is the tail (T)).^53^ For our system, we identify two critical dihedrals that will govern the aggregation behavior of the polymer chain: the backbone dihedral Θ_b_ between the thiophene rings of neighboring TBT units (Figure 1c) and the side chain dihedral Θ_s_ between the thiophene ring and the benzenethiol group within one monomer (Figure 1b). Details on the results of the reparametrization and the validation of the optimized force field can be found in Table S1 and Figures S1–S5 of the SI.

**Figure 2:**
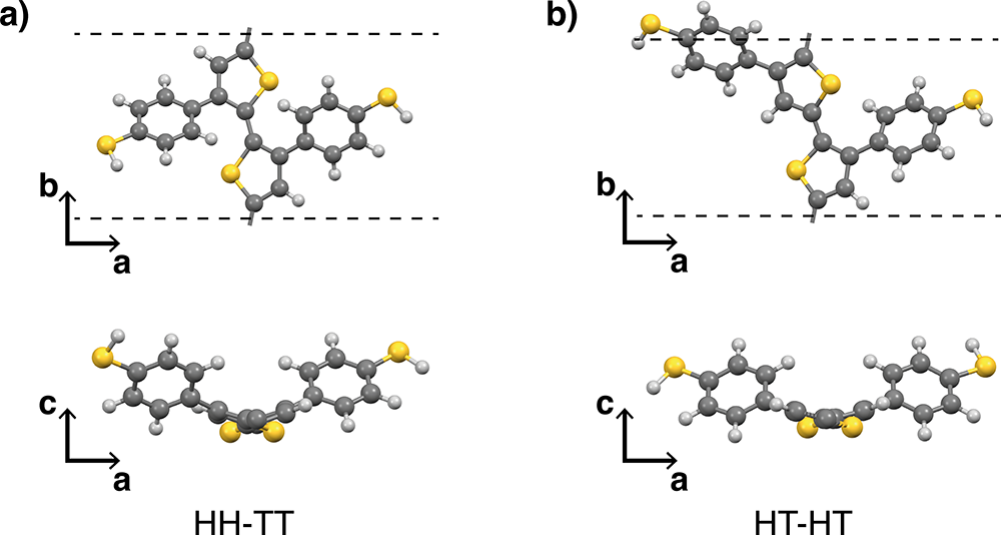
Illustration of the two possible regioregularities, (a) HH-TT and (b) HT-HT, of a conjugated PTBT chain. The upper two panels show the side view (*ab*-plane), and the lower two panels show the top view along the backbone direction (*ac*-plane). Dashed lines indicate the height of the unit cell along the *b*-direction.

In a real polymer system, on larger scales, both crystalline and amorphous domains will be present. As the first step in our investigation of charge transfer properties, we do not describe the polymer chains as oligomers but as periodically repeated chains of infinite length. Throughout the rest of this paper, we will apply periodic boundary conditions (PBC) along the axis of the polymer backbone. As a further simplification, we only consider regioregular (RR)-conjugated polymer chains,^46^ which follow a strict orientation of the alternating asymmetric repeating TBT units throughout the polymer backbone. This is, of course, an idealization of the real system in which the degree of regioregularity depends on synthetic conditions.^47,49,54^ Given the three possible connections between two TBT units (HH, HT, TT), there are two feasible RR-chains, namely HH-TT and HT-HT (cf. Figure 2).

We compare the structural properties of these regioregularities obtained from MD and DFT (cf. Table S2). Our results show that the presence of the benzenethiol groups leads to a deviation of the backbone planarity compared to the unsubstituted planar polythiophene chain.^55^ Such distortions from planarity have been observed for polythiophene systems substituted with similar bulky aromatic groups and can be related to steric effects between the side groups.^56,57^ For the HH-TT regularity, this is more prominent than for the HT-HT regularity because of the proximity of the benzenethiol groups in the HH intermonomer junction (cf. Figure S5, Table S2, and Supporting Text).

**Figure 3:**
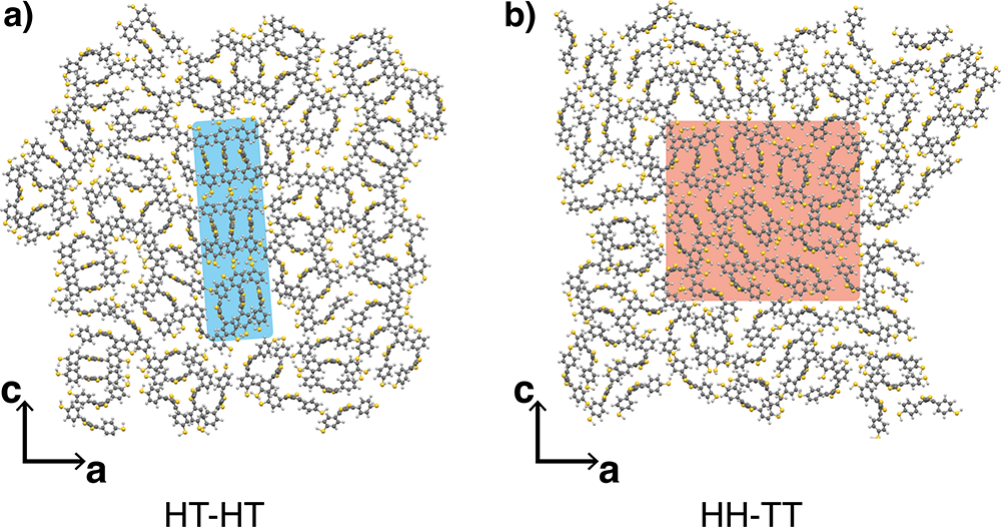
Comparison of the grown clusters at *T* = 300 K after annealing. The systems are shown in the *ac*-plane with the view along the polymer backbones. (a) The HT-HT system forms an ordered phase with a two-dimensional translational ordering along the *a*- and *c*-axes (blue-colored area). (b) The HH-TT system does not show long-range ordering in the *ac*-plane. For further investigation, we cut out a representative subset of the cluster’s center (red-colored area).

Next, we take the optimized single-chain structures to set up supercells of *N* = 100 PTBT chains for both regioregularities. Since there is no experimental data on the structural properties of this polymer present in the literature, we will simulate the self-assembly of conjugated PTBT chains from scratch. This means we let the system self-associate by temperature annealing from a very high temperature, isotropic phase to room-temperature conditions. Such an approach has been used successfully for similar systems.^58,59^ The initial systems are cooled down from 1500 to 300 K at a pressure of 1 bar in the *NPT* ensemble over a time period of 10 ns (cf. Figure S6). After the systems are equilibrated at room-temperature, all polymer chains have aggregated into one big cluster, respectively (cf. Figure 3). In the HT-HT system, we observe neighboring chains forming an ordered region in the center of the cluster, where the backbones are planar and stacked together in a face-to-face manner along the crystal (growth) *a*-axis. The blue-colored area depicts three stacks of chains arranged in a lamellar fashion along the *c*-axis. Going to the outer regions of such a cluster, its ordering decreases due to surface effects which lead to backbone bending and distortion of the crystalline alignment. Here, we observe a mix of different substructures (small stacks similar to the center, pairs of bent chains, and even bigger arrangements of those chain pairs; cf. Figure S6).

On the other hand, the HH-TT does not show ordered regions in the center of the grown cluster. Here, the backbones are still twisted, as is the case for the isolated chain. Thus, we see no stacking of multiple chains but rather arrangements of pairs. In contrast to the HT-HT system, we cannot observe any sign of long-range order, which suggests that the HH-TT polymer system cannot form crystalline phases. This can be related to the increased steric hindrance of the single chains making it harder for the HH-TT chains to adapt a planar configuration and thus aggregate into stacks.

In the following, we will investigate the structural and electronic properties of the two systems. For the HT-HT regularity, we will focus on the ordered phase without any surface effects. However, for HH-TT, this separation is not feasible due to its unordered behavior. Instead, we take a smaller subset of the cluster’s center as a representative system (red-colored area in Figure 3).

### Structural and Electronic Properties of the Crystalline (HT-HT) and Unordered (HH-TT) Phase

We start with the characterization of the HT-HT single-crystal by cutting a representative subset from the center of the grown cluster after the *NPT* annealing. This subset consists of 3 × 3 neighboring chains (blue colored area in Figure 3). From this, we create a periodic crystal by replicating the structures 4 × 4 times in the *ac*-plane. This homogeneous crystal, consisting of *N* = 144 polymer chains (12 × 12 in the *ac*-plane), is then equilibrated in another *NPT* run at 300 K for 5 ns. Afterward, a final *NPT* run for 1 ns is performed to collect structural data from the trajectory. The unit cell parameters, such as lattice lengths, angles, and mass density, are then averaged over the ensemble.

The results and their respective standard deviations due to temperature and pressure fluctuations are summarized in Table 1. We see that all angles differ from each other and do not contain the 90° angle typical for monoclinic crystals. Thus, the HT-HT forms a triclinic crystal structure at room-temperature. The crystal shows only minimal deviations from its equilibrium configuration due to thermal fluctuation (the standard deviation of the *a*- and *c*-axes is 0.3% and of the *b*-axis <0.1%).

**Table 1:** Crystallographic Data of the HT-HT Single-Crystal Phase from *NPT* Equilibration Simulations at *T* = 300 K and from DFT Optimization of the Primitive Cell^a^

	*a* (Å)	*b* (Å)	*c* (Å)	α (Å)	β (deg)	γ (deg)	ρ (g/cm^3^)
MD average	8.12(3)	7.68(1)	14.50(4)	92.5(2)	84.4(4)	108.4(4)	1.480(4)
DFT	7.44	7.78	14.45	89.2	89.7	90.2	1.51

a The first row denotes the block average over the ensemble of the 12 × 12 chains in the *ac*-plane with the standard deviation due to thermal fluctuations at room temperature in parentheses. The bottom row shows the DFT-optimized (PBE+MBD) results.

**Figure 4:**
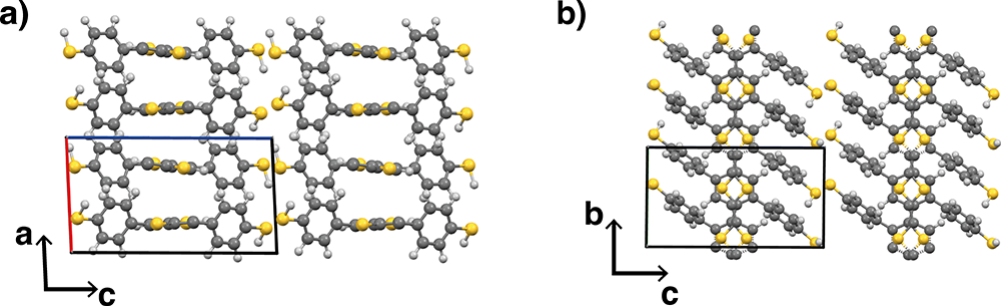
DFT optimized crystal structure of the HT-HT primitive unit cell. (a) The *ac*-plane and (b) *bc*-plane of the HT-HT crystal.

From the final *NPT* run, we take a snapshot of the homogeneous single-crystal, cut out the primitive unit cell, and optimize it with DFT at the PBE+MBD level of theory (bottom row of Table 1). The DFT optimization at 0 K mainly leads to a shortening of the *a*-axis by 8% corresponding to a tighter stacking of neighboring polymer backbones. This is also evident in the increased mass density (1.51 g/cm^3^ for the DFT compared to 1.48 g/cm^3^ for the MD run). We also notice that in the DFT structure, all angles are very close to 90° (with less than 1% deviation), suggesting an orthorhombic crystal. As already seen in the *NPT* run, in the crystal structure, the stacked chains now adopt a planar backbone conformation in contrast to the nonplanar isolated chains (Figure 4a). In Figure 4b, we see that the thiophene rings of neighboring chains (along the short stacking axis in *a*-direction) are not aligned on top of each other, but instead, they are shifted along the backbone direction (*b*-direction in Figure 4a) by one thiophene-thiophene distance. This creates space for the benzenethiol groups making the side chains from adjacent layers staggered and thus sterically less contentious. The thiol groups of neighboring stacks just touch each other, and there is no interdigitation of the side groups along the long stacking axis in *c*-direction.

**Figure 5:**
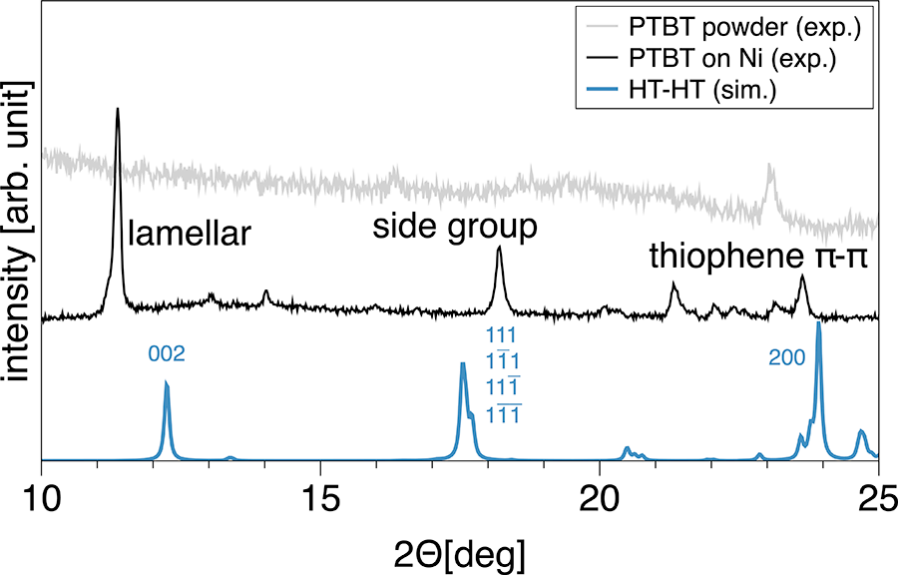
Comparison between the simulated XRD pattern for the theoretical crystal structure of HT-HT (blue) and two experimentally observed diffractograms for the PTBT film on a nickel foam (black) and as a powder (gray). For the simulated spectrum, Miller indices (*hkl*) are added to the main peaks.

In order to validate our structure prediction, we calculate XRD patterns based on the optimized HT-HT crystal and compare them to experimentally observed XRD diffractograms (cf. Figure 5). Measurements were performed on two different samples. The first sample is the PTBT polymer film deposited on a nickel surface, as it is prepared during the electropolymerization. With prolonged electropolymerization time, the PTBT film becomes thicker and looser, from which the outer layer is likely to fall off.^22^ The detached part of the PTBT polymer (called ‘PTBT powder’) was collected as the second sample. The PTBT polymer attached to the nickel surface (black line) shows strong diffraction with clearly observable crystal peaks. In contrast, the PTBT powder sample (gray line) shows no distinct peaks except one centered at 2Θ = 23°. For the ‘PTBT on Ni’ sample, we also observe strong peaks originating from the nickel itself (cf. Figure S9). Since these peaks occur at much higher Bragg angles (2Θ > 40°), they are easily distinguishable from the polymer signals. For the simulated diffractogram (blue line in Figure 5), we can assign Miller indices of the crystal lattice planes to the main peaks. Comparing the simulated pattern against that of the polymer attached to nickel allows us to distinguish three regions. The first peak of the measured pattern at around 2Θ = 11.4° is close to the secondary peak (002) of the theoretical one along the *c*-direction (long stacking axis). We, therefore, denote this as the lamellar peak. The next peak in the simulated diffractogram is a convolution of four different peaks originating from the planes defined by the four benzenethiol groups within the primitive cell (111, 11̅1, 111̅, and 11̅1̅). Hence, we suggest that the experimental peak at around 2Θ = 18.2° stems from the reflections of the side groups. Lastly, we observe several signals at higher Bragg angles with two pronounced peaks at 2Θ = 21.3° and 23.6° in the experimental spectrum. Using Bragg’s law *nλ* = 2*d* sin(Θ), this corresponds to distances of *d* = [3.7, 4.1] Å which are in the range of typical π–π stacking distances of similar polythiophene-based polymers.^60−62^ The simulated spectrum also features a distinct peak at 2Θ = 23.9°, corresponding to the stacking of thiophene backbones along the short *a*-axis. We can also relate the stacking distance *d* to the unit cell parameters by
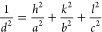
1with the Miller indices *hkl*. If we take the experimental peaks at 11.4° and 23.6° together with the Miller indices of the simulated pattern, we can get the lattice lengths *a* and *c*. The peak at 18.2° then gives us the lattice length *b*. From this, we calculate the following unit cell parameters based on the experimental diffractogram: *a* = 7.53 Å, *b* = 7.01 Å, and *c* = 15.56 Å. Compared to the DFT results (cf. Table 1), this gives deviations of 1.1%, 11.6%, and 7.2% (for *a*, *b*, and *c*, respectively).

The overall agreement with the simulated pattern suggests that the peaks of the PTBT-on-nickel sample mainly come from crystalline phases of chains with HT-HT regularity. The fact that the experimentally measured diffractogram can only be associated with the HT-HT crystal structure again emphasizes the importance of distinguishing between different regularities. Furthermore, the absence of crystallinity in the powder sample shows that, as the PTBT film becomes thicker during electropolymerization, its outer layers will lose most of its structural order. In the initial stage of electropolymerization, there is sufficient electrical contact between the polymer layers formed at the electrode surface. At this point, the polymer chains are short and efficient adhesion avoids twisting of the chains, forming higher regular and crystalline zones.^63^ As polymerization proceeds, large polymer chains tend to form disordered amorphous domains that affect the microstructure of the film. As the number of cycles increases, the film gradually becomes thicker, with a dense and rough surface.^64^ This is in agreement with similar electropolymerized thiophene-based polymers.^60,65,66^ Only the peak at 2Θ = 23° indicates that thiophene π–π stacking will remain to some degree in the PTBT-powder sample.

**Figure 6:**
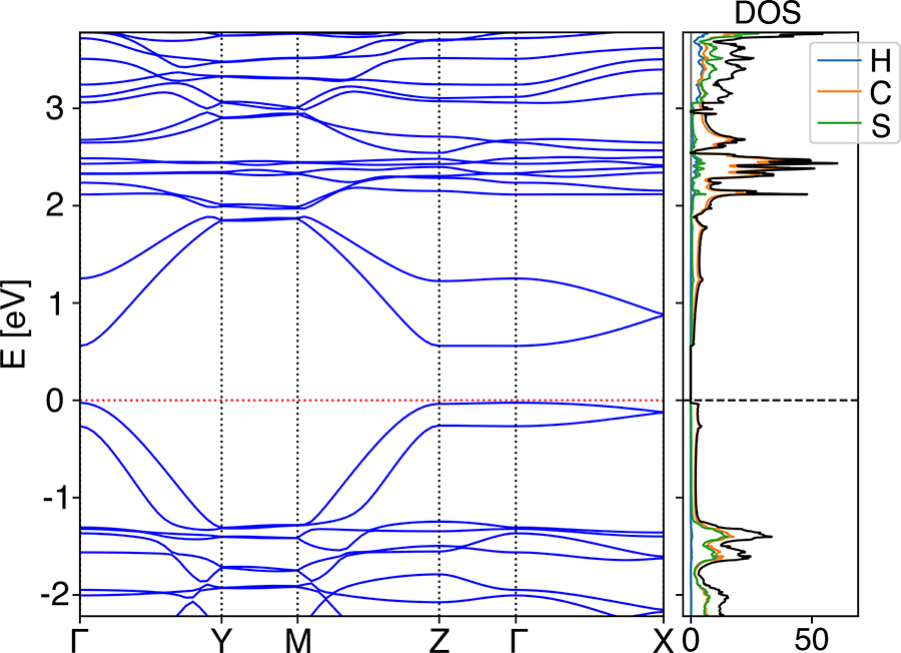
Band structure, DOS, and PDOS (hydrogen (H), carbon (C), and sulfur (S) are depicted with blue, orange, and green, respectively) for the HT-HT crystal. The band energies are shifted relative to the Fermi level. The Fermi level is indicated with a red dotted and gray dashed line.

Further, we looked at the electronic properties of the crystalline polymer phase. The band structure shows the HT-HT crystal to be a direct band gap semiconductor with a band gap of 0.58 eV at the Γ-point (cf. Figure 6). From the projected density of states (PDOS), we see that the shallow valence band and conduction band (CB) are mainly determined by the sp^2^-hybridized carbon atoms within the thiophene rings. In contrast, the thiophene sulfur atoms only contribute to the latter. The width of the CB along the Γ*Y* direction (direction of the conjugated backbones) is four times larger than that along the Γ*X* direction (short stacking direction) (1.29 and 0.31 eV, respectively). The bandwidth is almost zero in the direction of the long stacking axis (Γ*Z*). Large bandwidths are usually a characteristic feature of high mobility. Therefore, the band structure already suggests a two-dimensional charge transport with the main direction along the polymer backbones. Such a feature is typical for conjugated polymers which stack in a lamellar fashion.^40,41,67^

**Figure 7:**
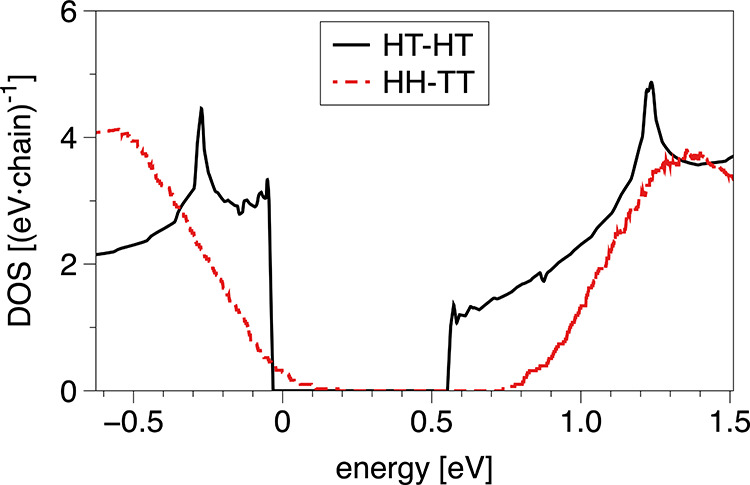
Comparison of the DOS of the crystalline HT-HT phase (black solid line) and the averaged DOS over five representative configurations of the unordered HH-TT phase (red dashed line).

In order to investigate the HH-TT system, we cut out a representative subset of the cluster’s center containing *N* = 23 polymer chains (cf. Figure 3b, red shaded area). This subsystem is then again equilibrated over 5 ns using an *NPT* simulation at *T* = 300 K. We randomly select five representative configurations for the electronic structure calculations from the last 10 ps of the equilibrated MD trajectory. The obtained MD structures are not further optimized with DFT but directly used due to the large system size. To estimate the band gap of the unordered HH-TT, we take an average over the values obtained from the five selected configurations. In Figure 7, the DOS of the crystalline HT-HT phase is compared with the averaged one of the selected HH-TT systems. As can be seen, the band gap of the disordered HH-TT phase (red dashed line) is larger by 24% than that of the crystalline HT-HT aggregates (black solid line), mainly due to a shift of the CB edge toward higher energies. The band gap is *E*_*g*_ = 0.72 ± 0.07 eV for the HH-TT system.

When comparing the systems in Figure 3, it can be seen that the polymer backbones in the HH-TT phase exhibit greater deviations from planarity than those in the ordered HT-HT phase (cf. also Figure S7 for a quantitative analysis), which indicates a break in conjugation in the former. It has already been observed for similar conjugated polymers that the band gap of their amorphous phases is much higher compared to that in well-ordered domains,^44,68^ which can be related to the reduced conjugation length (i.e., the length of periodicity^69^) along the polymer backbones of the amorphous phase. The DOS also displays a broadening of the tails near the band gap in the HH-TT system compared to the sharp band edges of the HT-HT phase. It is known that this broadening is caused by structural disorder, or so-called paracrystallinity,^70,71^ in the π-stacks of the system and the width of the DOS tails is a measure for the energetic disorder, that is, variations in the energy levels across the material.^68,72^

Furthermore, the broadening induces electronic states within the band gap of the crystalline HT-HT phase. It has been shown that disorder in the π-stacks of conjugated polymer chains causes the creation of deep tails of electronic states, which are much more localized than states inside of the bands.^37,73^ Thus, these states can act as traps for charge carriers, limiting their transport through the polymer.^74,75^ Beyond the static disorder of the HH-TT phase, dynamic disorder, that is, structural fluctuations over time, might be even more crucial in this domain as the relative position of neighboring chains will significantly influence their electronic coupling.^31^ The larger range of possible backbone dihedrals of HH-TT chains (cf. Figure S7) indicates an increased dynamic disorder in this phase compared to the HT-HT system.

Overall, the structural disorder in the HH-TT phase, caused by decreased packing efficiency, leads to an energetic disorder manifested in an enlarged band gap and the formation of potential trapping states within the band gap. In a heterogeneous microstructure where HT-HT and HH-TT phases coexist, the ordered HT-HT regions would be largely responsible for charge transport. If the band gap offset between the two phases is large enough so that the energetic overlap of electronic states vanishes, charges would be hindered from migrating between disordered and ordered regions.^68^ Charge carriers would then remain confined in the crystalline HT-HT phase.

Depending on the structural order of a material, one has to decide between different transport mechanisms. There is a wealth of literature on the topic of charge transport in molecular materials, but generally, one distinguishes two limiting regimes of band transport and charge hopping.^76^ Building on Bloch’s theorem,^77,78^ which describes very delocalized charge carriers, band-like approaches are by construction restricted to ordered and defect-free crystals. In our case, we will hence apply this ‘band’ picture only to the crystalline HT-HT phase. Given the morphological disorder of the HH-TT system, it is more appropriate to describe the charge transport herein either with a hopping model,^79,80^ assuming the charges to be localized on discrete sites or by trapping and releasing from localized states into higher energy mobile states.^81−83^ It should be noted that the models mentioned above still rely heavily on assumptions limiting their applicability, especially when it comes to intermediate cases of charge transport, which do not adhere to the limiting regimes.^76^ The development of methods^84−86^ that bridge the gap between these regimes to advance the understanding of charge transport in organic semiconducting materials is an ongoing field.

### Band-like Charge Transport in the HT-HT Phase

As a quantitative approach to estimate the intrinsic limits of charge transport in the PTBT polymer, we describe the charge transport in the HT-HT crystal within the band transport regime. The constant relaxation time approximation of the Boltzmann transport equation^87,88^ (eq 3) is employed to calculate the electrical conductivity and mobility. We first compute the relaxation time within the deformation potential theory (cf. eqs S2, S6, and S7) are used to calculate the deformation potential *D*_def,*ii*_ and the elastic constant *C*_*i*_ of the dilated unit cells along the three lattice directions (*i* = *a*, *b*, and *c*), respectively. With eq S8, we also compute the transport effective masses of the CB at its extrema. With these three parameters at hand, we obtain the electron/acoustic phonon scattering relaxation time τ_*ii*_ at 300 K.

From the results in Table 2, we see that the band structure’s anisotropy also manifests itself in the charge transfer properties. The flat CB along the *c*-axis (Γ*Z*) leads to a very high effective mass (*m*_eff_ = 96.12*m*_*e*_) compared to the other two directions with a modest (Γ*X*) and high band dispersion (Γ*Y*, cf. Figure 6). The elastic constant along the conjugated backbone in the *b*-direction is 1 order of magnitude higher than along the directions where the polymer chains are only bound by dispersive forces. Interestingly, the deformation potential, which describes the interaction strength of the charge carrier with the acoustic phonons, is largest for the transport along the *a*-axis with the tightly stacked thiophene backbones. The van der Waals interactions between neighboring chains which mainly govern the crystal structure in *a*- and *c*-directions are strongest in the direction of the π–π stacking of neighboring thiophene backbones. Thus, the total energy is more prone to structural deformations along the short stacking axis. This, in turn, leads then to a very short relaxation time along *a* of only a few femtoseconds. In comparison, the relaxation time along the conjugated backbone is 3 orders of magnitude higher, which shows that charge carriers can move relatively freely through the extended π-electron system along the backbone.

**Table 2:** Calculated Deformation Potential *D*_def,*ii*_ (in eV), Elastic Constant *C*_*i*_, Effective Transport Mass *m*_eff_, and Relaxation Time τ_*ii*_ along the Three Lattice Directions (*i* = *a*, *b*, and *c*) at 300 K

crystal direction *i*	*D*_def,*ii*_ (eV)	*C*_*i*_ (eV/Å)	*m*_eff_ (*m*_*e*_)	τ_*ii*_ (fs)
*a*	2.02	9.87	1.80	8.12
*b*	0.40	93.61	0.14	7.71 × 10^3^
*c*	0.14	9.76	96.12	2.37 × 10^2^

**Figure 8:**
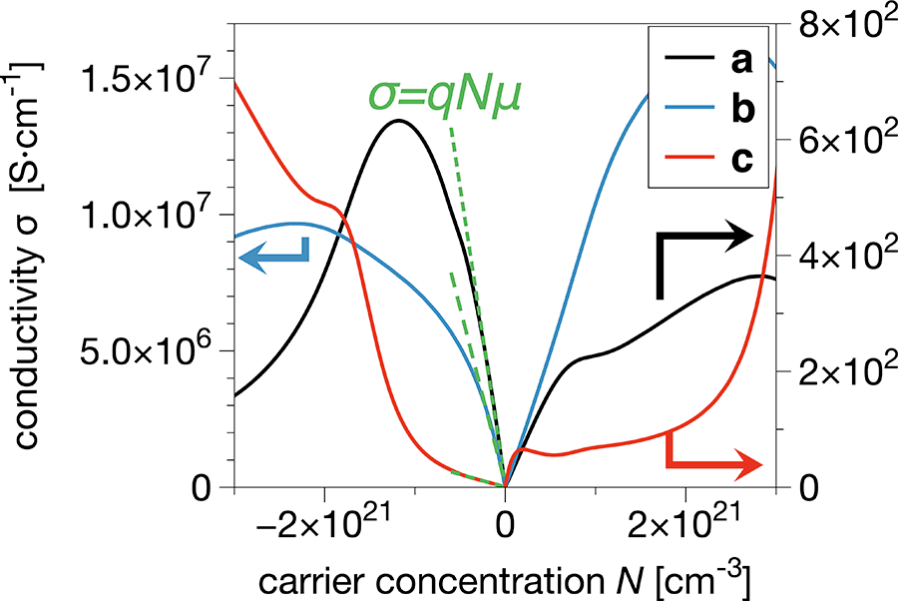
Plot of the band-like conductivity σ_*a*_ (black solid line), σ_*b*_ (blue solid line), and σ*_c_* (red solid line) along the *a*, *b*, and *c* directions, respectively, as a function of the charge carrier concentration *N* (the colored arrows associate the lines to their corresponding scales on the *y*-axis). The slope of the positive (negative) linear region of the conductivity over *N* gives the hole (electron) mobility according to eq 2 (green dashed lines, shown here for negative *N*).

With the band structure and the relaxation times, we can calculate the electrical conductivity σ in dependency of the charge carrier concentration *N* (by varying the chemical potential μ_chem_) according to eq 3. In Figure 8, we plot the conductivity σ_*a*_(*N*) (black solid line), σ_*b*_(*N*) (blue solid line), and σ_*c*_(*N*) (red solid line) along the *a*-, *b*-, and *c*-directions, respectively. As the chemical potential shifts, the dominant charge carriers change from electrons (negative *x*-axis) to holes (positive *x*-axis). A negative (positive) charge carrier concentration *N* resembles then n-type (p-type) doping of the polymer.

The electrical conductivity is apparently anisotropic. Along the conjugated backbones (*b*-direction), σ_*b*_ is 5 orders of magnitude larger than σ_*a*_ along the π–π stacking axis of the thiophene backbones and σ_*c*_ along the lamellar stacking (note the different scales on the *y*-axis in Figure 8). For a vanishing charge carrier concentration, we see that the intrinsic conductivity is zero in all cases, which is typical for semiconducting materials. Due to the system’s band gap, no thermally excited charge carriers contribute to conduction. This shows that an optimized doping state is crucial to guarantee the high conductivity of the system. Therefore, one would have to reduce the PTBT polymer to decrease or oxidate to increase the amount of doped anions. By comparing σ_*b*_ along the main transport direction for positive and negative *N*, we see that p-doping is favorable compared to n-doping since it leads to higher conductivities. This is the opposite for charge transport along the *a*- and *c*-directions.

Finally, we can determine the mobilities for charge transport along individual lattice directions by fitting the linear regions in Figure 8 according to eq 2. The mobility is given here by the slope of the linear fit. We calculate the electron mobilities as μ_*a*_ = 6.4 cm^2^/(V s), μ_*b*_ = 8.1 × 10^4^ cm^2^/(V s), and μ_*c*_ = 0.3 cm^2^/(V s). Again, the mobility along the polymer chains is much higher than in the other two directions. Although intrachain charge transport is expected to be faster than transport between different chains, the interchain charge transfer between backbones is known to be crucial for transport in a real polymeric system since macroscopic transport can not only be provided by single chains.^89,90^ The mobilities along the latter two directions are already at the lower end of the range where the picture of band-like charge transport is valid.^76^ Such anisotropic behavior has also been experimentally observed for polythiophene-based polymers.^91−93^ For instance, a high mobility anisotropy of μ_∥_/μ_⊥_ ≈ 1000 (where μ_∥_ and μ_⊥_ are the field-effect mobility along and orthogonal to the backbone orientation direction, respectively) was reported for highly oriented films of PBTTT.^94^ A similar anisotropy of the mobility of 4 × 10^3^ was found in poly(3-octylthiophene) single crystals.^95^ Yu et al. reported a mobility of up to 2.3 cm^2^/(V s) along the π-stacking direction of mesocrystalline P3HT.^96^ Luo et al. measured interchain mobilities along the π–π stacking direction of 1–2 cm^2^/(V s) for highly oriented nanocrystalline thiophene-based polymers.^91^ Overall, the agreement of our results with recent experimental values shows that a band-like description is able to explain the charge transport in well-ordered polymer systems.

As discussed, introducing disorder in crystalline structures can negatively affect their charge transport by introducing defects into the crystal lattice, causing domain boundaries and decreasing packing efficiency. Therefore, our results are an estimate of what might be ultimately achievable in a highly crystalline, defect-free polymer. An interesting aspect to investigate in future studies is to quantitatively assess the charge transport properties of the disordered phase as well. This, of course, necessitates an adequate theoretical model which accounts for any of the above-mentioned structural deviations from the well-ordered case. Another direction would be the band gap engineering of the system, for example, by explicit doping with ions to further improve the polymer’s transport properties.^97,98^

## Conclusions

In this study, we used a multiscale modeling approach to explore charge transport in conjugated organosulfur polymers for Li–S batteries. In particular, the focus has been set on the PTBT polymer, which has been extensively studied as an alternative cathode material.^22,25^ By using classical MD, for which we reparametrized important force field parameters based on DFT calculations, we simulated the self-assembled aggregation of polymer chains with different regioregularity. We found that the polymer chains can form crystalline phases only for a regioregular head-to-tail/head-to-tail (HT-HT) regularity, whereas for a head-to-head/tail-to-tail (HH-TT) regularity, the system did not show any long-range order after annealing. We can explain this structural difference by the increased steric constraints between neighboring TBT units within individual polymer chains in the HH-TT phase. Experimental XRD measurements confirmed the existence of crystalline phases in the electropolymerized PTBT, which we can relate to our predicted HT-HT crystal structure. We employed electronic structure theory to calculate the band structure and DOS of the different structural phases. It was observed that the polymer shows a semiconducting behavior. Further, we observed how the structural disorder in the HH-TT phase leads to an energetic disorder that can potentially limit the charge transport herein. Our calculations of conductivity as a function of charge carrier density offer a quantitative approach to estimating the intrinsic limit of the band-like mobility of the HT-HT phase along its crystal directions. Our results are in agreement with recent mobility measurements of similar crystalline thiophene-based polymers.

This study provides insights into the complex interplay between the microstructure and electrical properties of conjugated polymers, which serve as the cathode material for Li–S batteries. Specifically, the effects of polymer chain regioregularity on the morphology and the polymer’s electronic structure and charge transfer properties are demonstrated. Our work thus highlights the importance of polymer regularity and morphology modifications to design high-mobility crystalline phases, which would optimize the electrochemical performance of the cathode material. Work is in progress to extend our model to more realistic cathode structures and geometries, for example, including the influence of cross-linking sulfur between polymer chains as well as the solvent and electrolyte degrees of freedom. Hence, our study prepares future work on the described Li–S battery system.

## Methods

### Synthesis of PTBT

The monomer 4-(thiophen-3-yl)benzenethiol (TBT) was polymerized on a nickel foam (NF) with a thickness of 0.5 mm using a solution electrolyte of 0.1 M tetrabutylammonium hexafluorophosphate (TBAPF6) in acetonitrile (ACN). The electropolymerization process was carried out using a three-electrode system under potential range of −1.8–1.8 V (100 mV/s, *n* = 20) with an electrochemical workstation (GAMRY). An Ag wire was used as the reference electrode, and a platinum wire as the counter electrode. The resulting red-brown PTBT@NF electrode was obtained after rinsing the polymerized electrode with acetonitrile several times and drying it in a vacuum oven at 50 °C. During electropolymerization, the outer parts of the polymer detached from the electrode and were collected in the reaction vessel. After the polymerization reaction, the detached polymer was separated from the reaction medium (TBT and TBAPF6 in ACN) through 3 cycles of centrifugation using fresh acetonitrile to wash. After that, the remaining powdered PTBT polymer was dried in the same way as the one deposited on the nickel foam.

### XRD Characterization

X-ray diffraction (XRD) data were collected at Bruker D8 Advance for powder diffraction hosted by the HZB X-ray Corelab. The instrument makes use of a focusing X-ray beam consisting of characteristic copper wavelengths Kα_1+2_ and is equipped with a 1D LynxEye detector for fast powder diffraction data collection. Samples were prepared in a 9-fold sample flip-stick. Applied measuring time was 4 h per sample with a step size of 0.02° in the range of 10–130° for 2Θ. All measurements were carried out under ambient conditions.

### MD Simulations

Our systems are simulated on an *all-atom level* using the large-scale atomic/molecular massively parallel simulator package^99^ in combination with the OPLS force field.^100,101^ Details on the Hamiltonian of the force field and its adjustments regarding our specific system can be found in ref (48) and the Supporting Information (SI). The simulations are performed in an isothermal–isobaric (*NPT*) ensemble with periodic boundary conditions. The temperature and pressure are held constant with a Nosé–Hoover thermostat and barostat^102^ with a relaxation time of 100 and 1000 fs, respectively. The pressure is set to 1 bar.

We used a steepest descent algorithm to optimize the single chains. We also allowed the simulation box to relax in the periodic direction of the chains. In the other two directions, we set the box length to 40 Å to ensure a large distance between the periodic images of the chains. The aggregation process was explored using temperature annealing followed by an equilibrium simulation for a total period of 10 ns. To enhance the conformational sampling, the runs were started at an artificially high temperature of 1500 K and are then cooled down to 300 K (cooling rate of 0.12 K/ps). A time step of 1 fs was used in all simulations. The initial systems of *N* = 100 chains were set in orthogonal boxes, and during the run, we only applied the barostat to the *b*-direction of the box. After the initial *NPT* simulations, we took out representative subsets from the center of the resulting clusters to prepare another set of simulation cells. These systems were again equilibrated in an *NPT* ensemble simulation for 5 ns at *T* = 300 K. This time, we applied the barostat to all dimensions, and we also allowed the simulation boxes to adopt a triclinic shape.

### Electronic Structure Calculations

Electronic structure calculations are done at DFT level^103^ using the all-electron, full-potential density-theory package FHI-aims.^104−106^ The exchange-correlation interactions were treated using the Perdew–Burke–Enzerhof (PBE) functional^107^ together with the many-body dispersion (MBD) method^108^ to account for long-range van der Waals interactions. For comparison, we also used the hybrid PBE0 functional.^109^ The FHI-aims-specific tier 2 basis set and tight settings have been used. The convergence criterion for the total energy was set to 10^–5^ eV.

A Γ-centered *k*-grid of 1 × 8 × 1 was used for the optimization of the single chain structures and that of 8 × 8 × 4 for the optimization of the HT-HT crystal unit cell. The band structure of the HT-HT crystal was calculated on a finer *k* of 32 × 32 × 12. For the bigger HH-TT supercells, a coarse *k*-grid of 1 × 8 × 1 was used.

### Charge Transport Calculations

In general, the electrical conductivity of any material is determined by the concentration of mobile charge carriers *N* and their mobility μ by
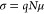
2where *q* is the charge of the carrier. We model the charge transport of PTBT in the crystalline HT-HT phase using the Boltzmann transport equation:^87,88^
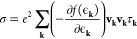
3where  is the Fermi–Dirac distribution function, **v**_**k**_ = ∇_**k**_ϵ_**k**_/*ℏ*, the group velocity of a charge carrier in a given band, ϵ_**k**_ is the band energy at a given **k**-point, ϵ_*F*_ is the Fermi-energy (the chemical potential at *T* = 0 K), *k*_B_ is the Boltzmann constant, and *T* the temperature. This equation describes the steady-state distribution of charge carriers in an electric field, considering the acceleration of the charges by the field and the restoration of the distribution through collisions with phonons and impurities.^110^ The so-called relaxation time τ_**k**_ describes the average time between these scattering events.^88^ In the crystalline phase of the polymer, we can neglect the influence of impurities and consider only scattering events with acoustic phonons,^88^ using the deformation potential theory^111^ to calculate the relaxation time along a crystal direction. The necessary parameters to assess the relaxation time can be determined by parabolic and linear fitting procedures.^112,113^ Together with the band structure of the system and for a given temperature, one can calculate the conductivity as a function of the charge carrier concentration by varying the chemical potential (more details on the derivation and the numerical schemes can be found in eqs S1–S11 of the SI).

## Supporting Information Available

Computational details of the charge transport property calculations. Force field dihedral reparametrization and validation. Structural properties of single PTBT chains. Aggregation behavior: Annealing and crystal growth. Structural analysis of the HT-HT and HH-TT phase. Calculation of the deformation potential and elastic constant. X-ray diffractogram of the PTBT polymer attached to nickel. Geometry of the optimized HT-HT crystal structure (PDF)

## Abbreviations

MD: molecular dynamics
DFT: density functional theory
XRD: X-ray diffraction
TBT: 4-(thiophene-3-yl)benzenethiol
PTBT: poly(4-(thiophene-3-yl)benzenethiol)
PBTTT: poly(2,5-bis(3-alkylthiophen-2-yl)thieno[3,2-b]-thiophene)
P3HT: poly(3-hexylthiophene)
OPLS: optimized potentials for liquid simulations
NPT: isothermal–isobaric ensemble
PBE: Perdew–Burke–Ernzerhof functional
PBE0: Perdew–Burke–Ernzerhof zero functional
MBD: many-body dispersion
HT: head-to-tail
HH: head-to-head
TT: tail-to-tail
HT-HT: head-to-tail/head-to-tail
HH-TT: head-to-head/tail-to-tail
DOS: density of states
PDOS: partial density of states
RBA: rigid band approximation
SP: single point
FF: force field
RB: Ryckaert–Bellmanns
PBC: periodic boundary conditions
RR: regioregular
