# Supplementary material for: How Regiochemistry Influences Aggregation Behavior and Charge Transport in Conjugated Organosulfur Polymer Cathodes for Lithium–Sulfur Batteries
Source: ACS Nano. 2023 Apr 4;17(8):7889–900. doi: 10.1021/acsnano.3c01523 (PMC10141565; doi:10.1021/acsnano.3c01523)
Supplement: Supplementary file 1 — nn3c01523_si_001.pdf [file nn3c01523_si_001.pdf]

# Supporting Information:

## How Regiochemistry Influences Aggregation Behavior and Charge Transport in Conjugated Organosulfur Polymer Cathodes for Lithium-Sulfur Batteries

Yannik Schütze,[†,‡] Diptesh Gayen,[¶] Karol Palczynski,[†] Ranielle de Oliveira Silva,[§] Yan Lu,[§,‖] Michael Tovar,[⊥] Pouya Partovi-Azar,[#] Annika Bande,[@] and Joachim Dzubiella[*,†,¶]

†*Research Group for Simulations of Energy Materials, Helmholtz-Zentrum Berlin für Materialien und Energie GmbH, Hahn-Meitner-Platz 1, 14109 Berlin, Germany*

‡*Theoretical Chemistry, Institute of Chemistry and Biochemistry, Freie Universität Berlin, Arnimallee 22, 14195 Berlin, Germany*

¶*Applied Theoretical Physics - Computational Physics, Physikalisches Institut, Albert-Ludwigs-Universität Freiburg, Hermann-Herder-Straße 3, 79104 Freiburg, Germany*

§*Department Electrochemical Energy Storage, Helmholtz-Zentrum Berlin für Materialien und Energie GmbH, Hahn-Meitner-Platz 1, 14109 Berlin, Germany*

‖*Institute of Chemistry, University of Potsdam, Am Neuen Palais 10, 14469 Potsdam, Germany*

⊥*Department Structure and Dynamics of Energy Materials, Helmholtz-Zentrum Berlin für Materialien und Energie GmbH, Hahn-Meitner-Platz 1, 14109 Berlin, Germany*

#*Institute for Chemistry, Martin Luther Universität Halle-Wittenberg, Von-Danckelmann-Platz 4, 06120 Halle (Saale), Germany*

@*Theory of Electron Dynamics and Spectroscopy, Helmholtz-Zentrum Berlin für Materialien und Energie GmbH, Hahn-Meitner-Platz 1, 14109 Berlin, Germany*

E-mail: joachim.dzubiella@helmholtz-berlin.de,joachim.dzubiella@physik.uni-freiburg.de

# Computational details of the charge transport property calculations

We model the charge transport of the PTBT in the crystalline phase with the Boltzmann transport equation (BTE).[S1,S2] The BTE expresses how a charge carrier distribution evolves in an electric field. The equation describes the steady-state situation between the acceleration of the charge by the external field, which drives the distribution out of equilibrium, and the collision with phonons and impurities, which restores the distribution.[S3] A common way to solve the BTE is the so-called relaxation time approximation. Here, one linearizes the relaxation of an electron distribution in a specific band, resulting in a time constant $\tau$ as the average time between scattering events that reset the charge distribution in this band.[S4] In the linearized version of the BTE, the electrical conductivity $\sigma$ can be expressed as

$$\sigma = e^2 \sum_{\mathbf{k}} \left( -\frac{\partial f(\epsilon_{\mathbf{k}})}{\partial \epsilon_{\mathbf{k}}} \right) \mathbf{v}_{\mathbf{k}} \mathbf{v}_{\mathbf{k}} \tau_{\mathbf{k}}. \tag{1}$$

Here, $f(\epsilon_{\mathbf{k}}) = 1/(e^{(\epsilon_{\mathbf{k}}-\epsilon_F)/k_B T} + 1)$ is the Fermi-Dirac distribution function, $\mathbf{v}_{\mathbf{k}} = \nabla_{\mathbf{k}} \epsilon_{\mathbf{k}}/\hbar$ the group velocity of a charge carrier in a given band, $\epsilon_{\mathbf{k}}$ the band energy at a given $\mathbf{k}$-point, $\epsilon_F$ the Fermi-energy (the chemical potential $\mu_{\text{chem}}$ at $T = 0$ K), $k_B$ the Boltzmann constant, and $T$ the temperature. We obtain the group velocities from the k-space derivatives of the quasi-particle energies given by the band structure. For this, we use the BoltzTraP2 software package, which applies a smoothed Fourier interpolation method to obtain an analytic expression for the band structure in order to calculate the $\mathbf{v}_{\mathbf{k}}$'s.[S5,S6] We interpolate the $\mathbf{k}$-grid of the self-consistent band structure calculation onto a five times finer grid to obtain converged results for the conductivity.

In eqn (1), the relaxation time $\tau_{\mathbf{k}}$ is energy- and $\mathbf{k}$-dependent. In general, the relaxation time accounts for scattering mechanisms such as elastic collisions of the charge carrier wave packet with impurities, as well as with optical and acoustic phonons.[S2] Since we apply the above theory only to the crystalline phase of the polymer, we can neglect any influence of

impurities. In such a molecular crystal, the coherence length of a delocalized charge carrier extends over several lattice constants. In this picture, the transport behavior of such a delocalized charge is mainly influenced by scattering events with acoustic phonons.[S7,S8] We here make use of the well-known deformation potential theory[S9] to calculate the relaxation time along a direction $i$ due to charge carrier-acoustic phonon interactions[S10]

$$\tau_{ii} = \frac{C_i \hbar^2}{\sqrt{k_B T m_{\text{eff},ii}} D^2_{\text{def},ii}}. \tag{2}$$

Three quantities are introduced in eqn (2). The deformation potential in direction $i$, $D_{\text{def},ii}$, is the response of the energy eigenvalue of the band in question at an extremal $\mathbf{k}$-point ($\mathbf{k_e}$) to a lattice deformation $\delta l_i$ along direction $i$

$$D_{\text{def},ii} = \left.\frac{\partial \epsilon(\mathbf{k})}{\partial \delta l_i}\right|_{\mathbf{k}=\mathbf{k_e}}. \tag{3}$$

As we aim to investigate the charge transport of electrons in our polymer, we are interested in the conduction band. The elastic constant of the crystal, $C_i$, is the response of the total electronic energy to a dilation in direction $i$

$$C_i = \frac{1}{l_i} \frac{\partial^2 E_{\text{tot}}}{\partial \delta l_i^2}, \tag{4}$$

where $l_i$ is the length of the respective lattice vector of the non-deformed crystal unit cell, and $E_{\text{tot}}$ is the total electronic energy of the unit cell. The third term is the effective mass, $m_{\text{eff,ii}}$, which, in its conventional definition, is given by the curvature of the band dispersion relation

$$m_{\text{eff},ii} = \frac{1}{\hbar^2} \frac{\partial^2 \epsilon(\mathbf{k})}{\partial k_i^2}. \tag{5}$$

Again, as we focus on electron transport, the reference point for the derivative is the conduction band minimum (CBM). Also notice the double indices in eqn (2), (3), and (5).

They occur because the relaxation time, in its general form, is a second-rank tensor. For simplicity, we assume a transformation into the principal axes of the crystal to only consider the diagonal elements.[S4] In order to obtain the deformation potential, we dilate (stretch and compress) the unit cell in all three Cartesian directions up to 3% and calculate the energy change of the conduction band with the lattice dilation. Since there is the inherent problem of DFT to calculate the absolute position of an energy level with respect to the vacuum level in an infinite periodic crystal, it is difficult to obtain absolute energy changes by comparing calculations with different lattice constants.[S7,S8] Therefore, we adopt an approximation by Wei and Zunger, which takes the energy of the lowest band as a reference to obtain absolute energy changes of the CBM, assuming that the 1s core level is not affected by small deformations of the unit cell.[S11] The deformation potential in eqn (3) is then fitted by

$$E_i^{\mathrm{CBM}} - E^{1\mathrm{s}} = D_{\mathrm{def},ii}\frac{\delta l_i}{l_i}. \tag{6}$$

For the elastic constant, the same stretching and compressing of the crystal must be done. The change of the total electronic energy is fitted to the cell dilation by[S11]

$$\frac{E_{\mathrm{tot}} - E_{\mathrm{tot}}^{\mathrm{eq}}}{l_i} = \frac{C_i}{2}\left(\frac{\delta l_i}{l_i}\right)^2, \tag{7}$$

with the total energy of the undeformed unit cell $E_{\mathrm{tot}}^{\mathrm{eq}}$ as the reference. Further, we use the EFFMASS package to obtain the effective mass for the different extremal points of the conduction band.[S12] In order to account for any non-parabolicity in the band structure, instead of eqn (5), we use a Kane quasi-linear dispersion relation[S13]

$$\frac{\hbar^2 k^2}{2m_{\mathrm{eff},ii}^t} = \epsilon(\mathbf{k})(1 + \alpha\epsilon(\mathbf{k})), \tag{8}$$

with the so-called transport effective mass $m_{\mathrm{eff},ii}^t$. In eqn (8), $\alpha$ accounts for the amount of non-parabolicity due to the flattening of an energy band (for $\alpha = 0$, one recovers the dispersion relation of a parabolic band).

With the band structure and the relaxation times, we can calculate the electrical conductivity at room temperature according to eqn. (1). Note that in the Fermi-Dirac distribution, the temperature $T$ and the chemical potential $\mu$ are external parameters. As we fix the temperature, the latter is still to be determined. For any real system, the chemical potential is determined by the experimental conditions applied during the synthesis (including defects and/or doping). This experimentally variable quantity cannot be captured within DFT. In a semiconducting material, the DFT would place the chemical potential (which at $T = 0$ K is the Fermi level) simply in the band gap to fulfill charge neutrality.

In general, the conductivity of any material is determined by the concentration of mobile charge carriers $N$ and their mobility $\mu$ by

$$\sigma = qN\mu, \tag{9}$$

with $q$ as the charge of the carrier. The carrier density can be separated into the intrinsic carrier density, which is due to thermally excited states of the band structure, and the carrier density originating from extrinsic contributions such as doping. The concentrations of electrons in the conduction band and holes in the valence band are given by[S14]

$$\rho_e = \int_{\mathrm{CB}} \mathrm{d}\epsilon \rho(\epsilon) f(\epsilon), \text{and} \tag{10}$$

$$\rho_h = \int_{\mathrm{VB}} \mathrm{d}\epsilon \rho(\epsilon)(1 - f(\epsilon)), \tag{11}$$

with the density of states (DOS) $\rho(\epsilon)$. The charge carrier concentration $N$ is the net concentration defined as the difference between electron and hole concentrations. Within the so-called rigid band approximation (RBA), we can model the effect of doping by simply changing the value of the chemical potential $\mu_{\mathrm{chem}}$ and, by this, the density of mobile electrons or holes. This assumes that changing $\mu_{\mathrm{chem}}$ does not affect the band structure. Moving $\mu_{\mathrm{chem}}$

into the conduction bands would lead to a higher electron density, resulting in an n-type material. Moving it into the valence bands would increase the hole density and produce a p-type material. Within the RBA, we then obtain the electrical conductivity as a function of charge carrier concentration by varying the value of the chemical potential.

## Force field dihedral re-parametrization and validation

The force field parameters we have used so far to describe the conjugated PTBT system[S15] using classical molecular dynamics simulation are taken from the original OPLS-AA force field.[S16] As this was the first approach to describe a new polymeric system, we aim to improve the description of our conjugated polymer further. In order to gain a better understanding of the crystallization behavior of the PTBT system, we need accurate dihedral energies. We, therefore, re-parametrize specific dihedral potentials by adopting the general force-field parametrization scheme by Wildman.[S17]

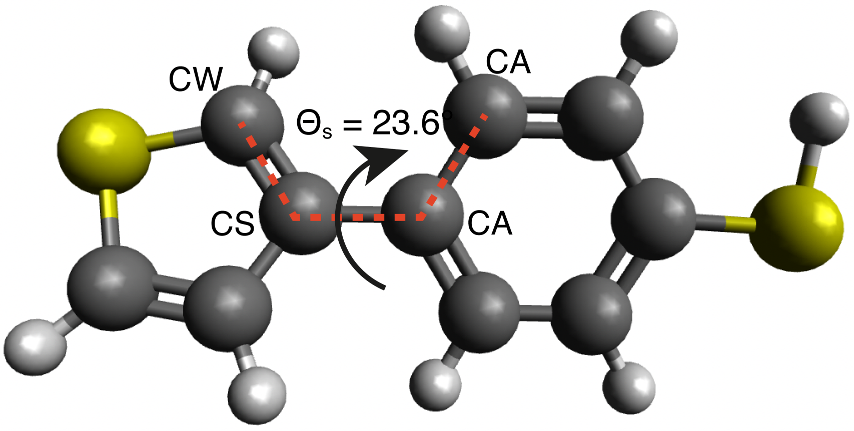


Figure S1: DFT optimized structure of a TBT monomer. The junction between the thiophene and benzene ring is governed by the side chain dihedral $\Theta_s$ (exemplified here by the atom set CW-CS-CA-CA). The most stable conformation features a dihedral angle of $\Theta = 23.6°$. Carbon, hydrogen, and sulfur elements are displayed as spheres in grey, white, and yellow, respectively.

First, we identify the critical dihedrals of our system. Starting with the basic repeating unit of the polymer (cf. Figure S1), we notice the bond between the thiophene ring and the benzenethiol group allows for a rotation of the two rings against each other. This junction is of special interest as the torsion between the rings will influence the orientation of $\pi$-orbitals and, therefore, the overall electronic structure and charge transfer properties. The DFT-optimized structure shows an inter-ring dihedral of $\Theta_s = 23.6°$.

In order to determine the dihedral energy parameters, we first scan the dihedral in steps of 10°, obtaining a series of relaxed molecular geometries. The geometry optimizations were carried out using the functional PBE. We then calculate the change of the total electronic energy $E_{\text{DFT}}$ of the whole system (encompassing all dispersive and electrostatic interactions between electrons and nuclei) in dependency of the dihedral angle $\Theta_s$. As a validation, we also adopted a two-step 'Scan - single point (SP)' approach[S18] where we used the hybrid PBE0 functional to calculate SP energies of the PBE-optimized structures (cf. Figure S2). The comparison shows a good agreement between the two functionals. Only the energies at the conjugation barrier (90°) appear different. This difference is $\sim 0.2$ kcal/mol, which at $\sim 7\%$ of the barrier itself is a negligible deviation. Given the similar results and the computational expense of using a hybrid functional, we chose PBE for all further calculations.

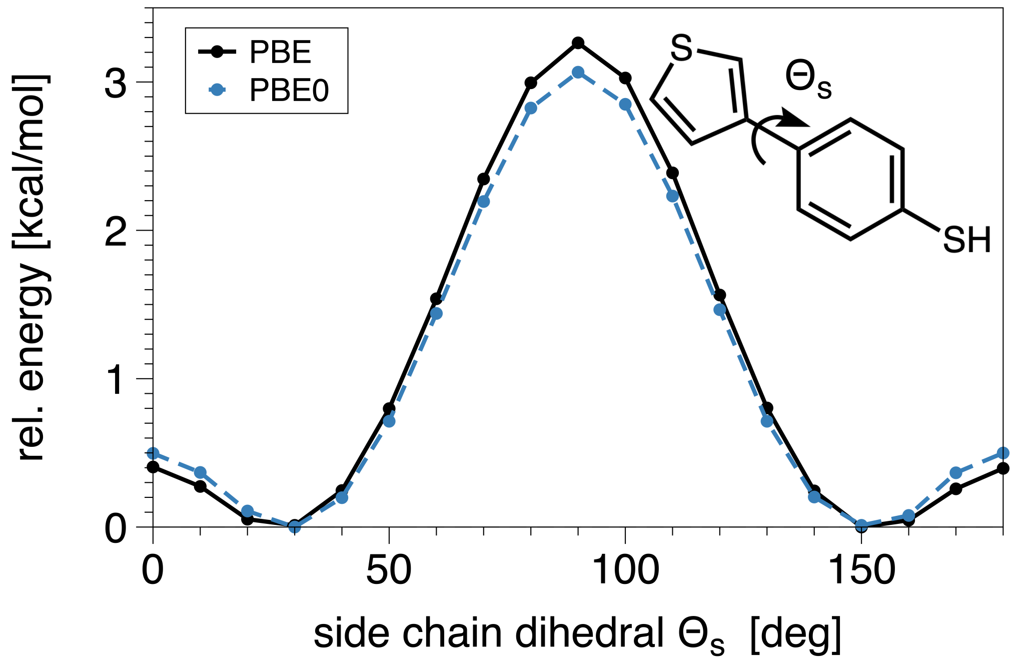


Figure S2: DFT scan of the TBT side chain dihedral $\Theta_s$ profile. All geometries were optimized with PBE (black solid line). In addition, we also calculated single point (SP) energies with the PBE0 functional (dashed blue line). The inset shows the molecular structure of a TBT monomer with the dihedral of interest.

Note that the DFT profile is not the same as the desired dihedral potential profile. In order to obtain the 'bare' dihedral profile, we have to isolate the energy of the dihedral from all other force field contributions. We, therefore, perform the same scan with our force field, but we set the energy terms of the dihedral to zero (see Figure S3, red solid line (FF)). If we then subtract the FF energy profile from the DFT profile, we obtain the effective dihedral potential and can fit it to a fifth-order Ryckaert-Bellmanns (dashed pink line (RB)) function[S19,S20]

$$V_{\mathrm{RB}}(\Theta) = \sum_{n=0}^{5} C_n \cos(\Phi)^n, \tag{12}$$

with $\Phi = \Theta - 180°$, where we adopt the 'polymer convention' casting the *trans* conformation at 0°.[S21] The 'complete' force field profile (dashed yellow line (FF + RB fit)) given by the addition of the FF scan and the fitted RB profile now matches the DFT profile to a

very high degree.

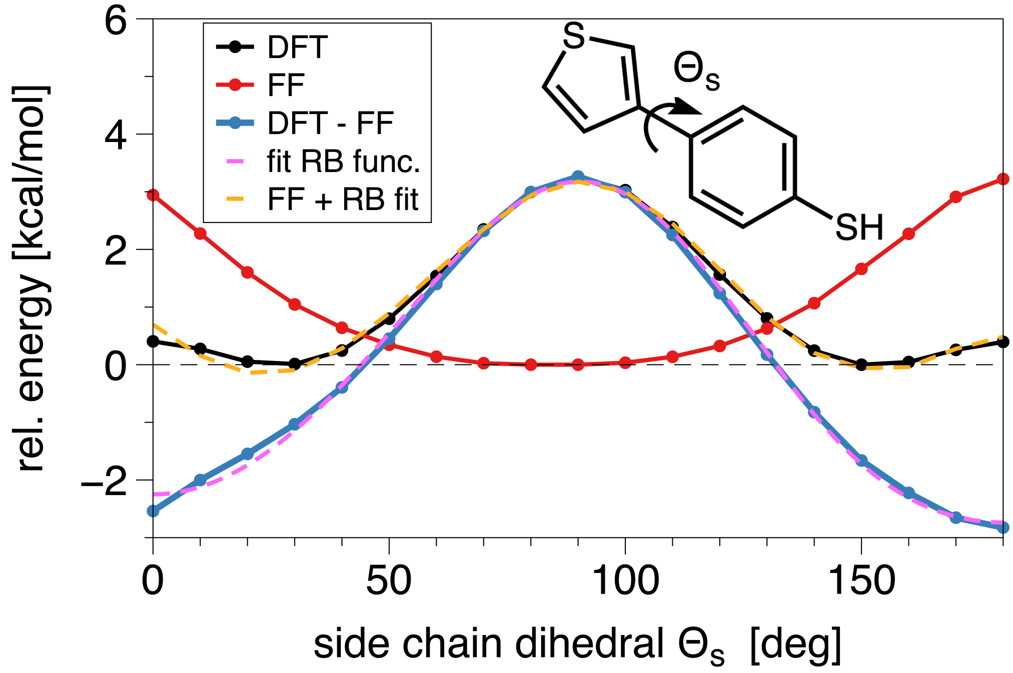


Figure S3: Illustration of the individual steps of the parametrization scheme for the inter-ring dihedral of one TBT monomer. The figure shows the DFT (PBE) profile (black), the profile obtained from the FF scan which excludes the dihedral contribution (red), the resulting substracted profile of the former two (blue), the fit of the subtracted profile to a fifth-order Ryckaert-Bellmans function (dashed pink), and the 'complete' profile given by the addition of the FF scan profile and the fitted profile (dashed yellow).

We now turn to the case of two neighboring, covalently bound TBT units. As explained in the main text, three possible connections (HH, HT, TT) can be formed between two TBT monomers during electro-polymerization (cf. Figure 1). Therefore, we must distinguish between these three configurations when parametrizing the dihedral potential $\Theta_b$ for the intermonomer junction. We apply the same procedure as for the TBT monomer to the parametrization of the three intermonomer junctions (cf. Figure S4). The force field parameters $C_n$ (cf. eqn. (12)) obtained from the above procedure are summarized in Table S1, in a form suitable for use with the LAMMPS MD package.[S22]

Table S1: Dihedral angle parameters $C_n$ (in kcal/mol) of the inter-ring torsion $\Theta_s$ within a TBT unit and the three possible intermonomer torsions $\Theta_b$ between two neighboring TBT units. The dihedral profiles were fitted with Ryckaert-Bellmanns functions according to eqn (12).

| dihedral angle | $C_0$ | $C_1$ | $C_2$ | $C_3$ | $C_4$ | $C_5$ |
|---|---|---|---|---|---|---|
| TBT | 0.794 | -0.030 | -1.892 | 0.334 | 0.474 | -0.243 |
| head-to-head (HH) | 0.410 | 1.058 | -0.356 | -2.008 | -0.904 | 0.643 |
| head-to-tail (HT) | 0.749 | 0.110 | -1.837 | -0.103 | 0.829 | 0.018 |
| tail-to-tail (TT) | 0.542 | 0.334 | -1.388 | -0.809 | 0.285 | 0.326 |

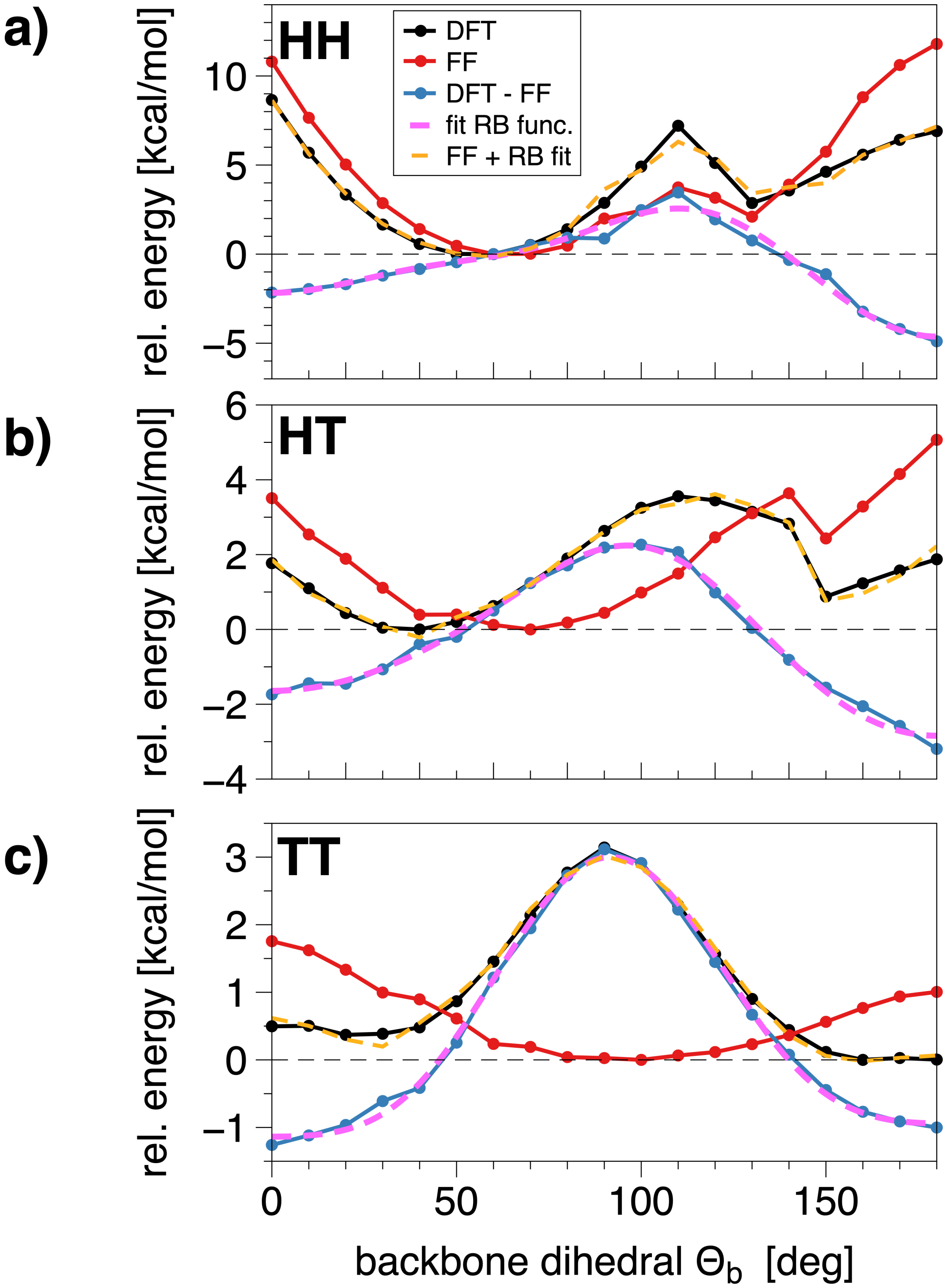


Figure S4: Illustration of the individual steps of the parametrization scheme for the three possible backbone dihedrals (HH, HT, TT). The figures show the DFT profile (black), the profile obtained from the FF scan which excludes the dihedral contribution (red), the resulting substracted profile of the former two (blue); the fit of the subtracted profile to a fifth-order Ryckaert-Bellmans function (dashed pink), and the 'complete' profile given by the addition of the FF scan profile and the fitted profile (dashed yellow). (Legend applies to all graphs).

In order to validate the performance of the re-parametrized force field, we compute the potential energy surface with respect to all four individual dihedrals and compare them with the DFT results (cf. Figure S5). The TBT side chain scan (upper panel) clearly shows that with the original parameters[S15] (dashed blue line), the energy barrier (90°) between the planar configurations (0° and 180°) was strongly overestimated by more than 22 kcal/mol. Because of this, the overall force field described a very rigid structure where the benzenethiol group had no flexibility to rotate but was kept at the planar conformation. With the improved 'new' parameters (red solid line), the energies match the DFT results (black solid line). For the three backbone dihedrals in the bottom figures, we additionally included calculations using only the improved side chain parameters while keeping the original backbone parameters (green dashed lines). This way, we can quantify how the adjustment of the side chain dihedral already improves the description of the backbone dihedral. For the HH case (second from above), we indeed see that adjusting the side chain already leads to a significantly improved backbone dihedral profile (green dashed line). This significant impact of the side chain can be explained by the proximity between the two benzenethiol groups in the HH structure (cf. Figure 1c). Due to their greater flexibility they can adapt more to the rotation of the backbone. This leads to less steric hindrance between the two monomers and, therefore, to a lowering of the total energy. The side chain's influence on the backbone's behavior is not as pronounced for the HT case as for the HH structure because the side groups are further apart. This is even more so for the TT case, as the benzenethiols are at the opposite ends of the dimer structure. Therefore, only adjusting the side chain dihedral does not improve the contribution of the backbone to the total energy (lower panel: the green dashed line still matches the blue dashed line). This fact underlines the importance of assessing both side chain and backbone dihedrals to improve the force field model.

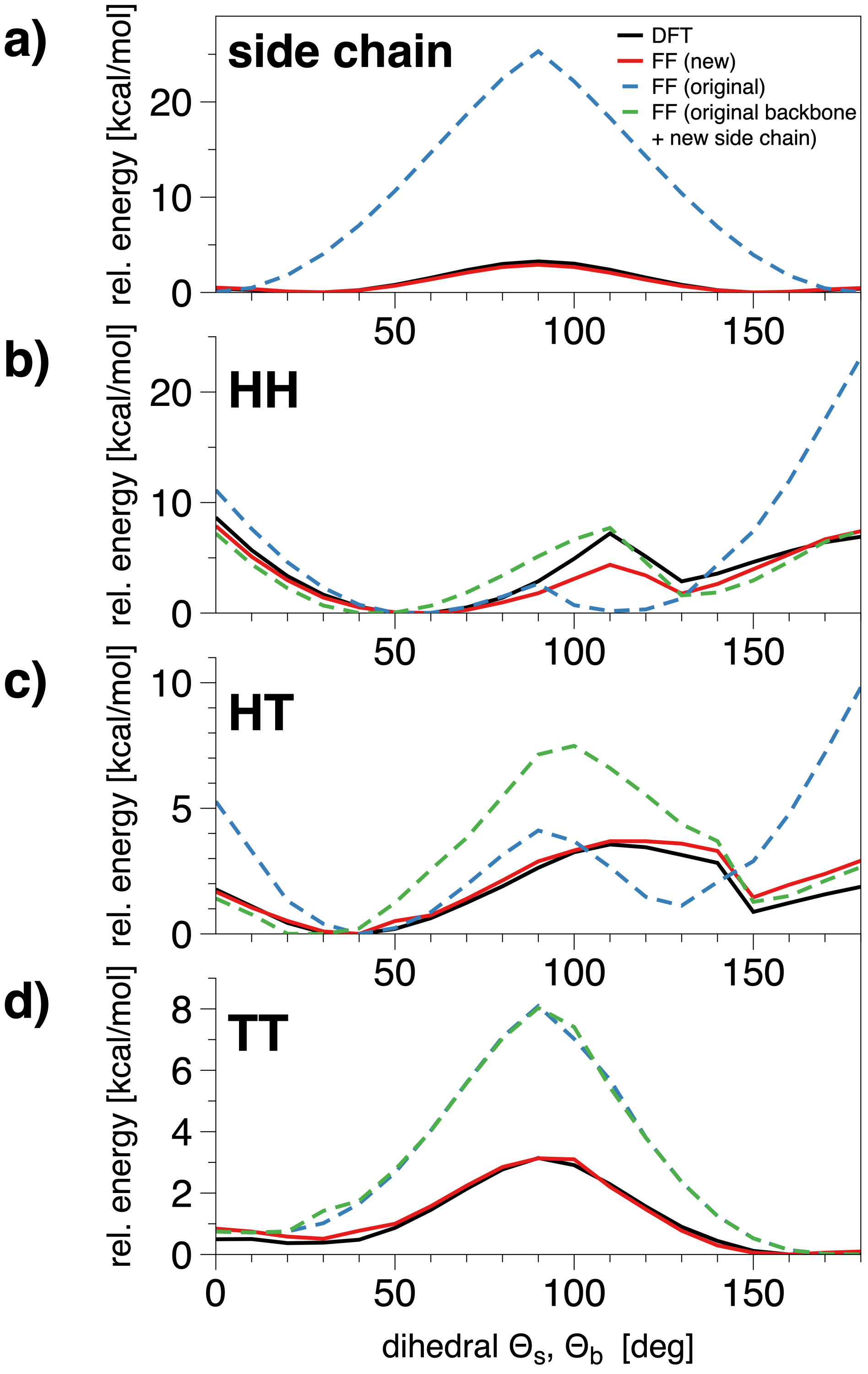


Figure S5: Comparison of the dihedral scans with the original force field[S15] (blue dashed line) and the improved 'new' force field of this study (red solid line) with the DFT results (black solid line). For the backbone dihedrals (HH, HT, TT), we also performed scans with the old backbone dihedral parameters in combination with the improved side dihedral parameters (green dashed line). (Legend applies to all graphs).

## Structural properties of single PTBT chains

We compare the results of the geometry optimization of the TBT monomer and two regioregular single chains between our force field and DFT. In Table S2, the structural properties of these three systems are summarized for the MD and two different DFT methods (PBE+MBD and PBE0+MBD). For better comparability of the dihedral angles, we report their deviation from planarity ($\Delta\Theta_{\mathrm{x}} = |180^\circ - \Theta_{\mathrm{x}}|$, with $x = \mathrm{s, b}$). For the TBT monomer, we compare the side chain dihedral. The periodic structures contain two monomers in the unit cell. Since two dihedrals constitute the chains, we have to consider two dihedrals for the side chain and backbone, respectively. For the dihedral angles, we obtain a maximum deviation of about $3-4^\circ$ between the MD and PBE0+MBD result, that is, approximately 10%. We also compare the height of the unit cell $L$, that is, the length of two repeating units along the chain between MD and DFT. Here, MD underestimates $L$ by roughly 1% compared to DFT. Comparing these structural features demonstrates that our classical force field accurately represents the monomeric and single-chain properties.

Comparing the two regularities, we notice that the two backbone dihedrals of an HT-HT chain are identical, whereas, in the case of an HH-TT chain, they differ by 15°. This is a consequence of the ordering of the side groups. In the HH-TT chain, the interaction of the benezenethiol groups between neighboring TBT units (HH) leads to a much higher deviation from planarity ($\Delta\Theta_{\mathrm{b}} = 41.4^\circ$ with PBE0) of the backbone than if the two hydrogens (TT) are adjacent ($\Delta\Theta_{\mathrm{b}} = 26.4^\circ$ with PBE0). Interestingly, both regularities are almost similar in their total electronic energy calculated by DFT (difference of $\delta E = 5$ meV with PBE0).

Table S2: Comparison of structural properties of an optimized TBT monomer and two isolated, periodic chains (HH-TT, HT-HT regularity). For the unit cell structures of the single chains, we have to consider two side chain and backbone dihedrals, respectively. For the backbone dihedral of the HH-TT chain, the upper value belongs to the HH case and the lower value to the TT case.

| model | TBT | HH-TT | | | HT-HT | | |
|---|---|---|---|---|---|---|---|
| | side chain $\Delta\Theta_s$ [deg] | side chain $\Delta\Theta_s$ [deg] | backbone $\Delta\Theta_b$ [deg] | $L^a$ [Å] | side chain $\Delta\Theta_s$ [deg] | backbone $\Delta\Theta_b$ [deg] | L [Å] |
| PBE | 27.9 | 47.3<br>46.5 | 37.3<br>19.5 | 7.78 | 55.5<br>55.5 | 22.3<br>22.1 | 7.80 |
| PBE0 | 28.9 | 46.7<br>45.2 | 41.4<br>26.4 | 7.75 | 53.5<br>53.9 | 28.3<br>28.4 | 7.76 |
| OPLS (MD) | 31.8 | 49.5<br>41.8 | 43.3<br>22.9 | 7.64 | 52.9<br>57.6 | 26.7<br>29.3 | 7.66 |

[a] $L$ is the height of the unit cell along the chain axis.

# Aggregation behavior: Annealing and crystal growth

To study the aggregation of PTBT polymer chains, we set up supercells of $N = 100$ chains for both regioregularities. Figure S6 shows exemplarily selected snapshots of the annealing process of the HT-HT system at several temperatures. As the temperature of the systems goes down, chains begin to aggregate and form many small clusters. Around a temperature of $T \approx 830$ K, chains start to stack together. Further cooling leads then to the growth of these aggregates by subsequent attachment of chains until these clusters eventually unite into a single large cluster. In the center of the cluster, neighboring chains form an ordered region with lamellar stacks (blue-colored area). In the outer regions of the cluster, the polymer chains have more freedom to bend due to thermal fluctuations. These surface effects lead to a distortion of the crystalline alignment where chains still form small stacks, but they also arrange in pairs of bent polymer chains. Interestingly, we can observe another substructure that almost looks like a micelle, in which chain pairs are arranged with all thiol groups pointing inward (black dashes line).

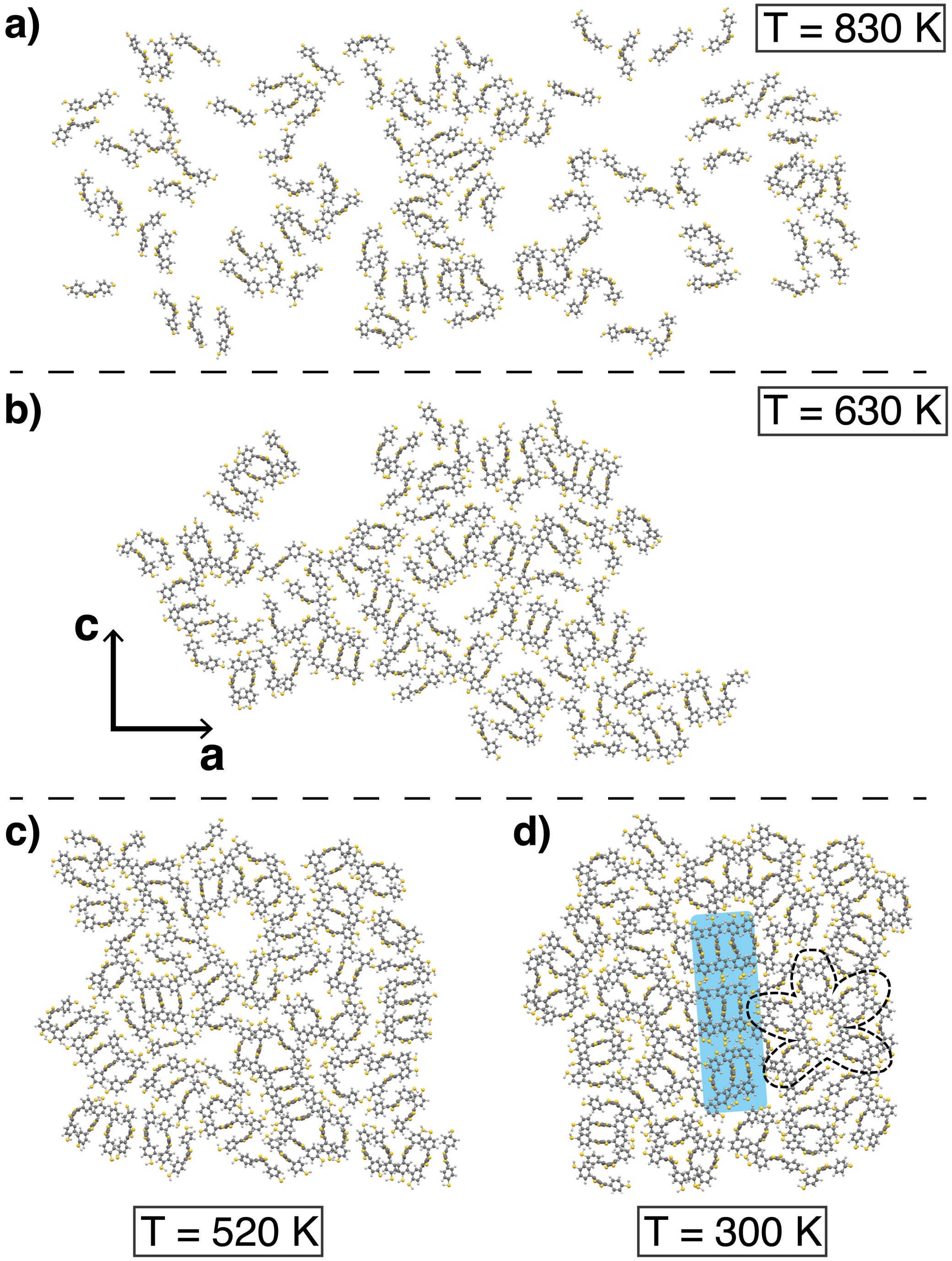


Figure S6: Snapshots of the aggregation process of the HT-HT PTBT polymer with respect to the annealing temperature $T$. (a) Pairs of chains start to stack together, with their backbones facing each other. (b) A big cluster starts to form. (c) The cluster becomes denser, with more and more chains forming stacks with their backbones aligning in a face-to-face manner. (d) A crystalline phase forms in the cluster's center with three stacks of chains arranged in a lamella (blue-colored area). Surface effects in the cluster's outer layers allow the chains to arrange in less ordered substructures, e.g., a micelle-like arrangement of chain pairs (black dashed line). All snapshots are shown in the $ac$-plane with the view along the polymer backbones.

## Structural analysis of the HT-HT and HH-TT phase

For both systems (HT-HT and HH-TT), we cut out representative subsets from the center of the grown clusters after the $NPT$ annealing (cf. section 4.2 in the main text for more details). For a quantitative analysis of the two phases, we sample the backbone dihedrals $\Theta_\mathrm{b}$ of all polymer chains over the last 10 ps of their respective $NPT$ runs. In Figure S7, we observe a clear difference in the distributions of backbone dihedrals of the two systems. As expected, the crystalline HT-HT (blue) shows one maximum at the planar backbone configuration ($\Theta_\mathrm{b} = 180°$). The system's finite temperature during the sampling ($T \approx 300$ K) causes thermal fluctuations of its equilibrium configuration, leading to a broadening of the distribution. On the other hand, the HH-TT system features two distinct and equally populated maxima, which deviate both by about 30° from the planar configuration. Furthermore, the total range of possible backbone dihedrals is much larger for the HH-TT than for the HT-HT phase.

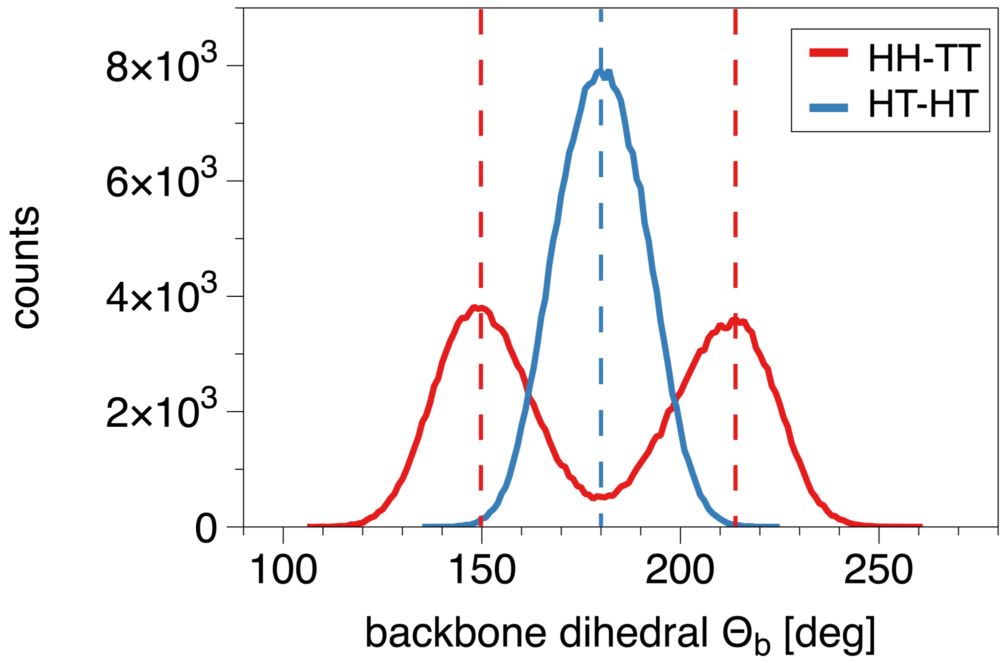


Figure S7: Distribution plot of the backbone dihedrals $\Theta_b$ of all polymer chains in the unordered HH-TT subset (red) and the homogeneous HT-HT crystal (blue) (cf. section 4.2 and Figure 3 in the main text for more details). The data was sampled from the last 10 ps of their respective *NPT* runs. The dashed lines serve as a guide to the eye to depict the maxima.

## Calculation of the deformation potential and elastic constant

In order to assess the relaxation time $\tau$ along the crystal directions of the HT-HT phase, we calculate the deformation potential and the elastic constant. As explained in the computational details, both parameter can be determined from a number of DFT geometry optimizations in stretched and compressed unit cells. Eq. (6) and (7) are then used to fit the change of the conduction band energy and the total energy to the lattice dilation, respectively (cf. Figure S8).

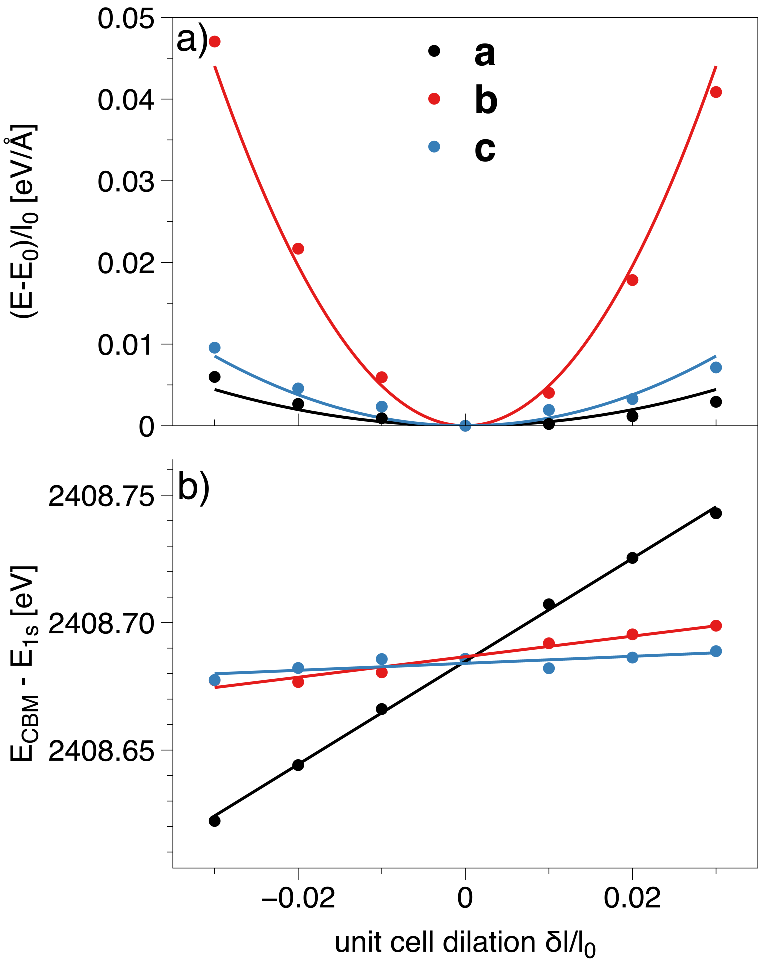


Figure S8: Numerical scheme to fit the elastic constant $C$ and the deformation potential $D_{\mathrm{def}}$. (a) The total electronic energy $E$ of the unit cell (referenced to the energy $E_0$ of the undeformed cell) in dependency of the dilation $\delta l/l_0$ along the lattice vectors **a**, **b**, and **c** (in black, red, and blue, respectively). The parabolic fit according to eqn. (S7) yields the elastic constant. (b) The shifts of the conduction band energy $E_{\mathrm{CBM}}$ (with respect to the lowest energy level $E_{1s}$) *versus* lattice dilation. The linear fits according to eqn. (S6) give the deformation potential as the slope.

# X-ray diffractogram of the PTBT polymer attached to nickel

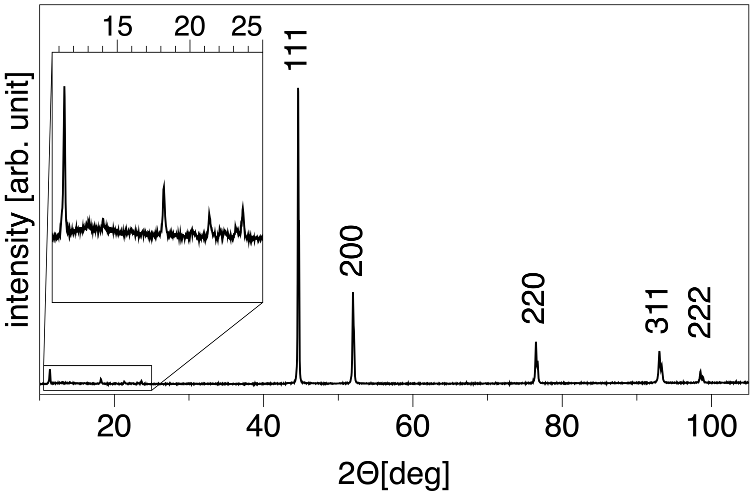


Figure S9: X-ray diffraction pattern of the PTBT film on a nickel (Ni) foam. The diffractogram shows the typical pattern of a face-centered cubic (fcc) phase of Ni (Miller indices are assigned to the main peaks).[S23,S24] The inset shows the signals of the PTBT polymer at lower Bragg angles.

# Geometry of the optimized HT-HT crystal structure

lattice_vector 7.43824238 0.08176976 0.03602299

lattice_vector -0.11706210 7.77782144 0.07550361

lattice_vector 0.00807419 0.07101866 14.45370871

atom 2.68768098 3.36657492 3.37504874 C

atom 1.70271259 3.07670674 5.64150195 C

atom 1.86134347 1.67775986 5.75817778 C

atom 1.98616530 1.03396834 4.88947463 H

atom 1.70968321 3.72399197 4.31916204 C

atom 0.72367183 4.64673790 3.93209401 C

atom 0.72164501 5.21459403 2.66080602 C

atom 1.71773505 4.86552848 1.73822444 C

atom -0.06871199 4.90859550 4.63161980 H

atom -0.06278777 5.91821429 2.38348835 H

atom 1.74017447 5.49681545 0.07885606 S

atom 1.32387768 6.76343752 0.36126138 H

atom 2.69780941 3.93298071 2.10443157 C

atom 1.71999455 6.40953693 6.22048101 S

atom 3.50406992 6.55881183 3.69928627 H

atom 1.50706164 5.06791475 7.30025791 C

atom 1.61798921 2.50323784 8.16379799 S

atom 1.68477118 7.57881616 7.51102870 C

atom 1.51422456 6.93918459 8.74377876 C

atom 1.80723838 1.18348928 7.05388277 C

atom 1.39084933 5.53956416 8.60184707 C

atom 1.23441645 4.88251369 9.45549664 H

atom 1.46638378 7.58273928 10.06787135 C

atom 2.61082679 0.65728739 10.42823569 C

atom 0.42146247 7.29418876 10.96079499 C

atom 0.51211563 0.06717563 12.16315818 C

atom 1.54088494 0.92409108 12.58168799 C

atom 7.05959877 6.70368100 10.68838424 H

atom 7.13144878 -0.09177053 12.87657911 H

atom 1.46188572 1.59564158 14.22211400 S

atom 2.59085906 1.22134752 11.70024066 C

atom 2.79930360 1.86246119 14.32439763 H

atom 1.55883965 3.69346447 6.89107667 C

atom 5.25054892 7.06213328 5.68826001 C

atom 5.13371595 5.65948872 5.80913109 C

atom 5.01576591 5.01123962 4.94277945 H

atom 5.21529581 7.70306849 4.36362769 C

atom 6.28173059 0.88294772 3.89419050 C

atom 4.25200374 7.30139958 3.42195215 C

atom 6.26294520 1.43956884 2.61833007 C

atom 4.33813655 0.07847290 2.07123873 C

atom 5.28280115 1.04380425 1.69761224 C

atom 7.06409005 1.18013385 4.59077964 H

atom 7.01991744 2.17061852 2.33589153 H

atom 5.24161292 1.65984344 0.03398588 S

atom 5.62794808 2.93792819 0.30930674 H

atom 3.46328228 2.65092843 3.64704244 H

atom 5.25084127 2.61849534 6.19454174 S

atom 3.56342770 -0.22639422 1.36852035 H

atom 3.48354430 3.66335720 1.39974872 H

atom 3.63186862 5.82183495 12.06259324 H

atom 3.61944436 4.83020825 9.80764361 H

atom 5.38970512 7.68517941 6.93531049 C

atom 3.44472663 0.87473483 9.76170328 H

atom 5.21753241 5.16869618 7.10448917 C

atom 5.26538430 3.78706737 7.48675997 C

atom 5.46506311 3.15061303 8.71704687 C

atom 5.51795441 1.28272524 7.26930633 C

atom 5.63130672 1.75584885 8.57045096 C

atom 5.81568896 1.10222728 9.42119661 H

atom 5.49523048 3.78683288 10.04494969 C

atom 4.45103327 4.61981033 10.47926563 C

atom 6.53461298 3.50399994 10.94596328 C

atom 4.44997516 5.17063190 11.75701661 C

atom 6.54047578 4.04220581 12.22949992 C

atom 5.49511602 4.87956540 12.64618514 C

atom 7.34720737 2.84533132 10.64034319 H

atom -0.08138457 3.72482467 12.87574954 H

atom 5.54437681 5.53656951 14.29360666 S

atom 4.19882213 5.76415371 14.38786845 H

atom 5.38517629 6.49509389 8.20996688 S

atom 3.39565349 1.88840525 12.00712721 H

The absolute energy of the optimized structure is −4736.90 Ha.

## References


(S1) Harris, S. *An Introduction to the Theory of the Boltzmann Equation*; Dover Publications: New York, 2004; pp 121–130.

(S2) Ziman, J. M. *Principles of the Theory of Solids*, 2nd ed.; Cambridge University Press: Cambridge, 1972; pp 211–230.

(S3) Licker, M.; Hill, M. *McGraw-Hill Concise Encyclopedia of Physics*, 1st ed.; McGraw-Hill Book Company: New York, 2005; pp 70–75.

(S4) Herring, C.; Vogt, E. Transport and Deformation-Potential Theory for Many-Valley Semiconductors with Anisotropic Scattering. *Phys. Rev.* **1956**, *101*, 944–961.

(S5) Madsen, G. K. H. Automated Search for New Thermoelectric Materials: The Case of LiZnSb. *Journal of the American Chemical Society* **2006**, *128*, 12140–12146.

(S6) Madsen, G. K.; Carrete, J.; Verstraete, M. J. BoltzTraP2, a Program for Interpolating Band Structures and Calculating Semi-Classical Transport Coefficients. *Comput. Phys. Commun.* **2018**, *231*, 140 – 145.

(S7) Tang, L.; Long, M.; Wang, D.; Shuai, Z. The Role of Acoustic Phonon Scattering in Charge Transport in Organic Semiconductors: A First-Principles Deformation-Potential Study. *Sci. China, Ser. B: Chem.* **2009**, *52*, 1646–1652.

(S8) Xi, J.; Long, M.; Tang, L.; Wang, D.; Shuai, Z. First-Principles Prediction of Charge Mobility in Carbon and Organic Nanomaterials. *Nanoscale* **2012**, *4*, 4348–4369.

(S9) Bardeen, J.; Shockley, W. Deformation Potentials and Mobilities in Non-Polar Crystals. *Phys. Rev.* **1950**, *80*, 72–80.

(S10) Beleznay, F. B.; Bogár, F.; Ladik, J. Charge Carrier Mobility in Quasi-One-Dimensional Systems: Application to a Guanine Stack. *Journal of Chemical Physics* **2003**, *119*, 5690–5695.

(S11) Wei, S.-H.; Zunger, A. Predicted Band-Gap Pressure Coefficients of All Diamond and Zinc-Blende Semiconductors: Chemical Trends. *Phys. Rev. B* **1999**, *60*, 5404–5411.

(S12) Whalley, L. D.; Frost, J. M.; Morgan, B. J.; Walsh, A. Impact of Nonparabolic Electronic Band Structure on the Optical and Transport Properties of Photovoltaic Materials. *Physical Review B* **2019**, *99*, 1–11.

(S13) Kane, E. O. Band Structure of Indium Antimonide. *Journal of Physics and Chemistry of Solids* **1957**, *1*, 249–261.

(S14) Kittel, C. *Introduction to Solid State Physics*, 8th ed.; John Wiley & Sons: New York, NY, 2004; pp 167–168.

(S15) Gayen, D.; Schütze, Y.; Groh, S.; Dzubiella, J. Solvation Structure of Conjugated Organosulfur Polymers for Lithium-Sulfur Battery Cathodes. *ChemRxiv,* 2023.

https://chemrxiv.org/engage/chemrxiv/article-details/63ea00621d2d1840636905f3 (accessed March 28, 2023).

(S16) Jorgensen, W. L.; Maxwell, D. S.; Tirado-Rives, J. Development and Testing of the OPLS All-Atom Force Field on Conformational Energetics and Properties of Organic Liquids. *Journal of the American Chemical Society* **1996**, *118*, 11225–11236.

(S17) Wildman, J.; Repiščák, P.; Paterson, M. J.; Galbraith, I. General Force-Field Parametrization Scheme for Molecular Dynamics Simulations of Conjugated Materials in Solution. *Journal of Chemical Theory and Computation* **2016**, *12*, 3813–3824.

(S18) Dubay, K. H.; Hall, M. L.; Hughes, T. F.; Wu, C.; Reichman, D. R.; Friesner, R. A. Accurate Force Field Development for Modeling Conjugated Polymers. *Journal of Chemical Theory and Computation* **2012**, *8*, 4556–4569.

(S19) Ryckaert, J.-P.; Bellemans, A. Molecular Dynamics of Liquid n-Butane Near Its Boiling Point. *Chemical Physics Letters* **1975**, *30*, 123–125.

(S20) Ryckaert, J.-P.; Bellemans, A. Molecular Dynamics of Liquid Alkanes. *Faraday Discuss. Chem. Soc.* **1978**, *66*, 95–106.

(S21) Chanda, M. *Introduction to Polymer Science and Chemistry: A Problem-Solving Approach*, 2nd ed.; Taylor & Francis: New York, 2013; pp 35–37.

(S22) Thompson, A. P.; Aktulga, H. M.; Berger, R.; Bolintineanu, D. S.; Brown, W. M.; Crozier, P. S.; in 't Veld, P. J.; Kohlmeyer, A.; Moore, S. G.; Nguyen, T. D.; Shan, R.; Stevens, M. J.; Tranchida, J.; Trott, C.; Plimpton, S. J. LAMMPS - A Flexible Simulation Tool for Particle-Based Materials Modeling at the Atomic, Meso, and Continuum Scales. *Comput. Phys. Commun.* **2022**, *271*, 108171.

(S23) Jović, V. D.; Maksimović, V.; Pavlović, M. G.; Popov, K. I. Morphology, Internal

Structure and Growth Mechanism of Electrodeposited Ni and Co Powders. *J. Solid State Electrochem.* **2006**, *10*, 373–379.

(S24) Wang, H.; Kou, X.; Zhang, J.; Li, J. Large Scale Synthesis and Characterization of Ni Nanoparticles By Solution Reduction Method. *Bull. Mater. Sci.* **2008**, *31*, 97–100.
